# List of primary types of the larentiine moth species (Lepidoptera: Geometridae) described from Indonesia - a starting point for biodiversity assessment of the subfamily in the region

**DOI:** 10.3897/BDJ.3.e5447

**Published:** 2015-07-17

**Authors:** Olga Schmidt

**Affiliations:** ‡ Zoologische Staatssammlung München, Munich, Germany

**Keywords:** Checklist, diversity, Eupitheciini, geometrid moths, Larentiinae, type specimens, Republic of Indonesia

## Abstract

**Background:**

The Indonesian geometrid moth fauna is rich and diverse, yet it is poorly studied. This is particularly the case for the second largest geometrid subfamily Larentiinae which comprises moths with predominantly high mountainous distribution in the tropics. The present study provides a first inventory of the primary type specimens of larentiine moth species (Lepidoptera: Geometridae) described from Indonesia.

**New information:**

The list of species described from Indonesia is arranged alphabetically by the tribe, genus, and species, and presents data on 251 species and subspecies. For each species type status, type locality, depository, and a full reference to the original description are listed. Synonyms with Indonesian type localities are included. The study indicates a large part of the Indonesian geometrid fauna belong to the tribe Eupitheciini.

## Introduction

Indonesia is a large archipelago, comprising thousands of islands in Southeast Asia and Oceania, with a total area of about 1,91 million square kilometers. It shares land borders with Malaysia, East Timor and Papua New Guinea, and maritime boundaries with the neighbouring countries including Australia, Palau, Philippines, Singapore, Thailand, Vietnam and the Indian territory of the Andaman and Nicobar Islands. Although several surveys of the rich and diverse fauna of Macrolepidoptera have been conducted in selected regions of Indonesia (*e.g.* Heppner 1989, Barlow and Woiwod 1990, Sutrisno 2005, Sutrisno 2008, Sutrisno 2009), checklists of species comprising geometrid moths are far from being complete despite taxonomic information being essential for understanding biodiversity, distribution of species, and ecosystem structure. For instance, recent accounts of geometrid species for an area in Central Kalimantan by Sutrisno (2005) and Beck and Rüdlinger (2014), include four and five larentiine species, respectively. A comprehensive list of West Papuan Larentiinae, including not yet confirmed Papua New Guinea species, is presented on the website by de Vos (2014), although the species list needs to be checked and updated according to recent literature data. It is necessary to mention that sorting of specimens for biodiversity surveys was often undertaken on a visual basis in the absence of adequate identification tools (see Barlow and Woiwod 1990) and without comparison with type specimens which reduces the reliability of species identifications.

Larentiinae are the second most species-rich geometrid subfamily after Ennominae, with 6,230 described species (Scoble and Hausmann 2007) and numerous undescribed species. The present study aims to create an inventory of larentiine species (Lepidoptera, Geometridae) with Indonesian type localities, providing original references. A special emphasis on primary types excludes inaccuracies caused by incorrect identification, and the list provides a starting point for an assessment of the biodiversity of the subfamily Larentiinae in Indonesia.

## Materials and methods

The present list brings together scattered knowledge on the larentiine moths described from Indonesia. Major taxonomic papers embracing data on the Lepidoptera fauna of Indonesia were checked, from the earliest records by Walker (1863) until the latest reports by Schmidt (2005), Schmidt (2006b), including a comprehensive catalogue of Geometridae by Scoble (1999) and using a list of references by Gressitt and Szent-Ivany (1968). In addition, geometrid moth collections of the Natural History Museum, London, UK (NHM), the Indonesian Institute of Sciences, Cibinong, Indonesia (LIPI), Museum für Naturkunde der Humboldt-Universität zu Berlin, Germany (MNHU), Royal Belgian Institute of Natural Sciences, Brussels (RBINS) and SNSB-Zoologische Staatssammlung München (ZSM) were examined, revealing undescribed species and stressing the need of further taxonomic studies. Other abbreviations are: f – female; m – male; Mt – mount; NBC – Naturalis Biodiversity Center, Leiden, the Netherlands; NHRS – Naturhistoriska Riksmuseet, Stockholm, Sweden; OUM – Oxford University Museum of Natural History, UK; ZMMU – Zoological Museum, Moscow State University, Russia.

The name of the original genus is given in parentheses after the name of the valid genus. The status of the type is noted. Citations of references for each species are given under "Nomenclature". The altitude is presented as in the original description. Valid species, valid subspecies and synonyms with Indonesian type localities are included. "Distribution" embraces the type locality only.

## Checklists

### List of primary types of the larentiine moth species described from Indonesia

#### Asthenini

#### Bihastina (Bihastina) albolucens

Prout, 1916

Bihastina (Bihastina) albolucens Prout 1916

##### Materials

- **Type status:** Holotype. **Occurrence:** sex: m; **Record Level:** ownerInstitutionCode: NHM

##### Distribution

Type locality: [West Papua], Dutch New Guinea

##### Notes

The species is illustrated in Xue and Scoble (2002)

#### Parasthena (Parasthena) flexilinea

Warren, 1902

Parasthena (Parasthena) flexilinea Warren 1902

##### Materials

- **Type status:** Syntype. **Occurrence:** sex: 5m, 5f; **Record Level:** ownerInstitutionCode: NHM

##### Distribution

Type locality: Celebes [Sulawesi], Bonthain, 3000-7000 ft.

##### Notes

The species is illustrated in Holloway (1997) and in Xue and Scoble (2002)

#### Poecilasthena (Poecilasthena) limnaea

Prout, 1926

Poecilasthena (Poecilasthena) limnaea Prout 1926

##### Materials

- **Type status:** Syntype. **Occurrence:** sex: 2m, 2f; **Record Level:** ownerInstitutionCode: NHM

##### Distribution

Type locality: [West Papua], Mt Goliath, about 139° E, 5000-7000 ft.

#### Cidariini

#### Dysstroma (Polyphasia) ceprona

(Swinhoe 1902)

Dysstroma (Polyphasia) ceprona Swinhoe 1902

##### Materials

- **Type status:** Syntype. **Occurrence:** sex: f; **Record Level:** ownerInstitutionCode: NHM

##### Distribution

Type locality: Sumatra

#### Dysstroma (Polyphasia) cuneifera

(Warren 1898)

Dysstroma (Polyphasia) cuneifera Warren 1898

##### Materials

- **Type status:** Holotype. **Occurrence:** sex: m; **Record Level:** ownerInstitutionCode: NHM

##### Distribution

Type locality: Java, Mt Arjuno

#### Ecliptopera (Ecliptopera) ctenoplia ctenoplia

Prout, 1931

Ecliptopera (Ecliptopera) ctenoplia ctenoplia Prout 1931

##### Materials

- **Type status:** Holotype. **Occurrence:** sex: m; **Record Level:** ownerInstitutionCode: ZMMU

##### Distribution

Type locality: Java, Tjibodas

#### Ecliptopera (Ecliptopera) ctenoplia rectificata

Prout, 1940

Ecliptopera (Ecliptopera) ctenoplia rectificata Prout 1940

##### Materials

- **Type status:** Syntype. **Occurrence:** sex: 1m, 1f; **Record Level:** ownerInstitutionCode: NHM

##### Distribution

Type locality: Bali (east), Git-Git

#### Ecliptopera (Ecliptopera) odontoplia

Prout, 1935

Ecliptopera (Ecliptopera) odontoplia Prout 1935

##### Materials

- **Type status:** Syntype. **Occurrence:** sex: 5m, 6f; **Record Level:** ownerInstitutionCode: NHM

##### Distribution

Type locality: Java (east), Singolangoe

#### Ecliptopera (Ecliptopera) rectilinea rectilinea

Warren, 1894

Ecliptopera (Ecliptopera) rectilinea rectilinea Warren 1894

##### Materials

- **Type status:** Syntype. **Occurrence:** sex: many; **Record Level:** ownerInstitutionCode: NHM

##### Distribution

Type locality: Syntype localities: India, Khasia Hills, Celebes (south) [Sulawesi]

##### Notes

The species is described from India, Khasia Hills and Indonesia, Celebes (south) [Sulawesi]. The species is illustrated in Holloway (1997)

#### Ecliptopera (Ecliptopera) rectilinea fortis

Prout, 1932

Ecliptopera (Ecliptopera) rectilinea fortis Prout 1932a

##### Materials

- **Type status:** Syntype. **Occurrence:** sex: 2m; **Record Level:** ownerInstitutionCode: RBINS

##### Distribution

Type locality: Celebes [Sulawesi], Tonsea Lama

#### Ecliptopera (Ecliptopera) rectilinea impingens

Prout, 1937

Ecliptopera (Ecliptopera) rectilinea impingens Prout 1937

##### Materials

- **Type status:** Syntype. **Occurrence:** sex: 3m; **Record Level:** ownerInstitutionCode: NHM

##### Distribution

Type locality: Bali (west), Mondoktoempang, 2500 ft.

#### Ecliptopera (Ecliptopera) thalycra

Prout, 1928

Ecliptopera (Ecliptopera) thalycra Prout 1928

##### Materials

- **Type status:** Holotype. **Occurrence:** sex: m; **Record Level:** ownerInstitutionCode: NHM

##### Distribution

Type locality: Sumatra, Slopes of Mt Korintji, 7300 ft.

#### Electrophaes (Electrophaes) fulgidaria chrysodeta

Prout, 1928

Electrophaes (Electrophaes) fulgidaria chrysodeta Prout 1928

##### Materials

- **Type status:** Holotype. **Occurrence:** sex: m; **Record Level:** ownerInstitutionCode: NHM

##### Distribution

Type locality: Sumatra, Slopes of Mt Korintji, 7300 ft.

#### Eupitheciini

#### Antimimistis (Antimimistis) attenuata melamphaes

Prout, 1958

Antimimistis (Antimimistis) attenuata melamphaes Prout 1958

##### Materials

- **Type status:** Holotype. **Occurrence:** sex: unknown; **Record Level:** ownerInstitutionCode: NHM

##### Distribution

Type locality: Celebes [Sulawesi], Paloe, G. Tompoe, 2700 ft.

##### Notes

*The species A. attenuata* (Moore, 1887) is illustrated in Holloway (1997)

#### Ardonis (Chloroclystis) filicata mochleutes

(Prout 1958)

Ardonis (Chloroclystis) filicata mochleutes Prout 1958

##### Materials

- **Type status:** Holotype. **Occurrence:** sex: m; **Record Level:** ownerInstitutionCode: NHM

##### Distribution

Type locality: Celebes [Sulawesi], Tjamba, near Maros, 1500 ft.

##### Notes

The species *A. filicata* (Swinhoe, 1892) is illustrated in Holloway (1997)

#### Ardonis (Chloroclystis) thaumasta

(Prout 1935)

Ardonis (Chloroclystis) thaumasta Prout 1935

##### Materials

- **Type status:** Holotype. **Occurrence:** sex: m; **Record Level:** ownerInstitutionCode: NHM

##### Distribution

Type locality: Java (east), Tengger, Kletak, 6000 ft.

#### Axinoptera (Chloroclystis) melampepla

(Prout 1958)

Axinoptera (Chloroclystis) melampepla Prout 1958

##### Materials

- **Type status:** Holotype. **Occurrence:** sex: f; **Record Level:** ownerInstitutionCode: NHM

##### Distribution

Type locality: Celebes (west) [Sulawesi], Paloe, G. Rangkoenau, 1800 ft.

#### Bosara (Chloroclystis) atypha

(Prout 1958)

Bosara (Chloroclystis) atypha Prout 1958

##### Materials

- **Type status:** Holotype. **Occurrence:** sex: m; **Record Level:** ownerInstitutionCode: NHM

##### Distribution

Type locality: Celebes (west) [Sulawesi], Paloe, G. Rangkoenau, 1800 ft.

#### Bosara (Chloroclystis) catabares

(Prout 1958)

Bosara (Chloroclystis) catabares Prout 1958

##### Materials

- **Type status:** Holotype. **Occurrence:** sex: unknown; **Record Level:** ownerInstitutionCode: NHM

##### Distribution

Type locality: Celebes (west) [Sulawesi], Paloe, G. Tompoe, 2700 ft.

#### Bosara (Chloroclystis) cuneativenis

(Prout 1958)

Bosara (Chloroclystis) cuneativenis Prout 1958

##### Materials

- **Type status:** Holotype. **Occurrence:** sex: unknown; **Record Level:** ownerInstitutionCode: NHM

##### Distribution

Type locality: Celebes (west) [Sulawesi], Paloe, G. Tompoe, 2700 ft.

#### Bosara (Bosara) dilatata pelopsaria

Walker, 1866

Bosara (Bosara) dilatata pelopsaria Walker 1866a

##### Materials

- **Type status:** Syntype. **Occurrence:** sex: f; **Record Level:** ownerInstitutionCode: OUM

##### Distribution

Type locality: [Moluccas], Sula

##### Notes

The species *B. dilatata* Walker (1866) is illustrated in Holloway (1997)

#### Bosara (Gullaca) festivata

(Warren 1903)

Bosara (Gullaca) festivata Warren 1903

##### Materials

- **Type status:** Syntype. **Occurrence:** sex: 2f; **Record Level:** ownerInstitutionCode: NHM

##### Distribution

Type locality: Celebes [Sulawesi]

##### Notes

The species is illustrated in Holloway (1997)

#### Calluga (Micrulia) crassitibia

(Warren 1901)

Calluga (Micrulia) crassitibia Warren 1901a

##### Materials

- **Type status:** Holotype. **Occurrence:** sex: m; **Record Level:** ownerInstitutionCode: NHM

##### Distribution

Type locality: Barat Daya Islands, Dammer [Damar] island

#### Calluga (Calluga) grammophora

Prout, 1958

Calluga (Calluga) grammophora Prout 1958

##### Materials

- **Type status:** Holotype. **Occurrence:** sex: m; **Record Level:** ownerInstitutionCode: NHM

##### Distribution

Type locality: [West Papua], Mt Goliath, 5000-7000 ft.

#### Calluga (Calluga) psaphara

Prout, 1929

Calluga (Calluga) psaphara Prout 1929b

##### Materials

- **Type status:** Holotype. **Occurrence:** sex: m; **Record Level:** ownerInstitutionCode: NHM

##### Distribution

Type locality: [Moluccas], Buru, Wa'Katin, 1675 ft.

#### Chloroclystis (Chloroclystis) analyta

Prout, 1928

Chloroclystis (Chloroclystis) analyta Prout 1928

##### Materials

- **Type status:** Holotype. **Occurrence:** sex: m; **Record Level:** ownerInstitutionCode: NHM

##### Distribution

Type locality: Sumatra, Slopes of Mt Korintji

#### Chloroclystis (Chloroclystis) inaequata scotosema

Prout, 1937

Chloroclystis (Chloroclystis) inaequata scotosema Prout 1937

##### Materials

- **Type status:** Syntype. **Occurrence:** sex: 11m, 11f; **Record Level:** ownerInstitutionCode: NHM

##### Distribution

Type locality: Bali (east), Batoeriti, 3500 ft.

#### Chloroclystis (Chloroclystis) palmaria palmaria

Prout, 1928

Chloroclystis (Chloroclystis) palmaria palmaria Prout 1928

##### Materials

- **Type status:** Holotype. **Occurrence:** sex: m; **Record Level:** ownerInstitutionCode: NHM

##### Distribution

Type locality: Sumatra, Slopes of Mt Korintji

#### Chloroclystis (Chloroclystis) palmaria phantastes

Prout, 1958

Chloroclystis (Chloroclystis) palmaria phantastes Prout 1958

##### Materials

- **Type status:** Holotype. **Occurrence:** sex: m; **Record Level:** ownerInstitutionCode: NHM

##### Distribution

Type locality: Java, Gedeh, 7500 ft.

#### Chloroclystis (Chloroclystis) permixta

Prout, 1958

Chloroclystis (Chloroclystis) permixta Prout 1958

##### Materials

- **Type status:** Holotype. **Occurrence:** sex: unknown; **Record Level:** ownerInstitutionCode: NHM

##### Distribution

Type locality: Java, Tengger, Kletak, 6000 ft.

#### Chloroclystis (Chloroclystis) semiscripta brychoma

Prout, 1958

Chloroclystis (Chloroclystis) semiscripta brychoma Prout 1958

##### Materials

- **Type status:** Holotype. **Occurrence:** sex: unknown; **Record Level:** ownerInstitutionCode: NHM

##### Distribution

Type locality: Celebes (west) [Sulawesi], Paloe, Rangkoenau

##### Notes

The species *C. semiscripta* Warren (1906) is illustrated in Holloway (1997)

#### Chloroclystis (Chloroplintha) velutina

(Warren 1897)

Chloroclystis (Chloroplintha) velutina Warren 1897a

##### Materials

- **Type status:** Syntype. **Occurrence:** sex: 2m; **Record Level:** ownerInstitutionCode: NHM

##### Distribution

Type locality: Celebes (south) [Sulawesi], Bonthain, 5000-7000 ft.

#### ‘Chloroclystis’ (Chloroclystis) acervicosta

Prout, 1958

‘Chloroclystis’ (Chloroclystis) acervicosta Prout 1958

##### Materials

- **Type status:** Holotype. **Occurrence:** sex: m; **Record Level:** ownerInstitutionCode: NHM

##### Distribution

Type locality: Lesser Sunda Islands, Sambawa [Sumbawa]

#### ‘Chloroclystis’ (Chloroclystis) alpnista eupora

Prout, 1958

‘Chloroclystis’ (Chloroclystis) alpnista eupora Prout 1958

##### Materials

- **Type status:** Holotype. **Occurrence:** sex: f; **Record Level:** ownerInstitutionCode: NHM

##### Distribution

Type locality: Bali (west), Mondoktoempang, 2500 ft.

#### ‘Chloroclystis’ (Chloroclystis) apotoma

Prout, 1958

‘Chloroclystis’ (Chloroclystis) apotoma Prout 1958

##### Materials

- **Type status:** Holotype. **Occurrence:** sex: m; **Record Level:** ownerInstitutionCode: NHM

##### Distribution

Type locality: SW Celebes [Sulawesi], G. Lampobattang, Parang-bobo Goa, 5000 ft.

#### ‘Chloroclystis’ (Chloroclystis) autopepla

Prout, 1958

‘Chloroclystis’ (Chloroclystis) autopepla Prout 1958

##### Materials

- **Type status:** Holotype. **Occurrence:** sex: m; **Record Level:** ownerInstitutionCode: NHM

##### Distribution

Type locality: [West Papua], Mt Goliath, 5000-7000 ft.

#### ‘Chloroclystis’ (Chloroclystis) boarmica

Prout, 1958

‘Chloroclystis’ (Chloroclystis) boarmica Prout 1958

##### Materials

- **Type status:** Holotype. **Occurrence:** sex: f; **Record Level:** ownerInstitutionCode: NHM

##### Distribution

Type locality: SW Celebes [Sulawesi], G. Lampobattang, Parang-bobo Goa, 5000 ft.

#### ‘Chloroclystis’ (Chloroclystis) dentatissima

Warren, 1898

‘Chloroclystis’ (Chloroclystis) dentatissima Warren 1898

##### Materials

- **Type status:** Syntype. **Occurrence:** sex: 19, mostly females; **Record Level:** ownerInstitutionCode: NHM

##### Distribution

Type locality: [Moluccas], Key [Kai] Islands

#### ‘Chloroclystis’ (Chloroclystis) distigma

Prout, 1958

‘Chloroclystis’ (Chloroclystis) distigma Prout 1958

##### Materials

- **Type status:** Holotype. **Occurrence:** sex: f; **Record Level:** ownerInstitutionCode: NHM

##### Distribution

Type locality: [West Papua], Snow Mts, Upper Setekwa River, 2000-3000 ft.

#### ‘Chloroclystis’ (Gymnoscelis) inops

(Warren 1898)

‘Chloroclystis’ (Gymnoscelis) inops Warren 1898

##### Materials

- **Type status:** Holotype. **Occurrence:** sex: m; **Record Level:** ownerInstitutionCode: NHM

##### Distribution

Type locality: [Moluccas], Key [Kai] Islands

#### ‘Chloroclystis’ (Chloroclystis) invisibilis invita

Prout, 1958

‘Chloroclystis’ (Chloroclystis) invisibilis invita Prout 1958

##### Materials

- **Type status:** Holotype. **Occurrence:** sex: m; **Record Level:** ownerInstitutionCode: NHM

##### Distribution

Type locality: Celebes (west) [Sulawesi], Koelawi, Paloe, 3700 ft.

#### ‘Chloroclystis’ (Chloroclystis) leucopygata cata

Prout, 1958

‘Chloroclystis’ (Chloroclystis) leucopygata cata Prout 1958

##### Materials

- **Type status:** Holotype. **Occurrence:** sex: unknown; **Record Level:** ownerInstitutionCode: NHM

##### Distribution

Type locality: SW Celebes [Sulawesi], Pangean near Maros, 2000 ft.

#### ‘Chloroclystis’ (Chloroclystis) manusela

Prout, 1929

‘Chloroclystis’ (Chloroclystis) manusela Prout 1929b

##### Materials

- **Type status:** Syntype. **Occurrence:** sex: 2f; **Record Level:** ownerInstitutionCode: NHM

##### Distribution

Type locality: [Moluccas], Ceram (central) [Seram], Manusela

#### ‘Chloroclystis’ (Chloroclystis) phoenicophaes

Prout, 1958

‘Chloroclystis’ (Chloroclystis) phoenicophaes Prout 1958

##### Materials

- **Type status:** Holotype. **Occurrence:** sex: unknown; **Record Level:** ownerInstitutionCode: NHM

##### Distribution

Type locality: Celebes (west) [Sulawesi], Paloe, G. Tompoe, 2700 ft.

#### ‘Chloroclystis’ (Rhinoprora) rufitincta

(Warren 1898)

‘Chloroclystis’ (Rhinoprora) rufitincta Warren 1898

##### Materials

- **Type status:** Syntype. **Occurrence:** sex: 1m, 5f; **Record Level:** ownerInstitutionCode: NHM

##### Distribution

Type locality: Java, Mt Arjuno

#### ‘Chloroclystis’ (Chloroclystis) solidifascia

Prout, 1929

‘Chloroclystis’ (Chloroclystis) solidifascia Prout 1929b

##### Materials

- **Type status:** Syntype. **Occurrence:** sex: 2f; **Record Level:** ownerInstitutionCode: NHM

##### Distribution

Type locality: [Moluccas], Ceram (central) [Seram], Manusela, 6000 ft.

#### ‘Chloroclystis’ (Chloroclystis) speciosa

Swinhoe, 1902

‘Chloroclystis’ (Chloroclystis) speciosa Swinhoe 1902

##### Materials

- **Type status:** Syntype. **Occurrence:** sex: f; **Record Level:** ownerInstitutionCode: NHM

##### Distribution

Type locality: [West Papua], Kapaur

#### ‘Chloroclystis’ (Chloroclystis) taraxichroma

Prout, 1958

‘Chloroclystis’ (Chloroclystis) taraxichroma Prout 1958

##### Materials

- **Type status:** Holotype. **Occurrence:** sex: m; **Record Level:** ownerInstitutionCode: NHM

##### Distribution

Type locality: Bali (east), Batoeriti, 3500 ft.

#### ‘Chloroclystis’ (Chloroclystis) viridata phaeina

Prout, 1958

‘Chloroclystis’ (Chloroclystis) viridata phaeina Prout 1958

##### Materials

- **Type status:** Holotype. **Occurrence:** sex: f; **Record Level:** ownerInstitutionCode: NHM

##### Distribution

Type locality: Celebes (west) [Sulawesi], Paloe, G. Tompoe, 2700 ft.

#### ‘Chloroclystis’ (Chloroclystis) xenisma

Prout, 1958

‘Chloroclystis’ (Chloroclystis) xenisma Prout 1958

##### Materials

- **Type status:** Holotype. **Occurrence:** sex: m; **Record Level:** ownerInstitutionCode: NHM

##### Distribution

Type locality: Celebes (west) [Sulawesi], Paloe, G. Tompoe, 2700 ft.

#### Chrysoclystis (Chrysoclystis) perornata

Warren, 1896

Chrysoclystis (Chrysoclystis) perornata Warren 1896

##### Materials

- **Type status:** Syntype. **Occurrence:** sex: 3f; **Record Level:** ownerInstitutionCode: NHM

##### Distribution

Type locality: [West Papua], Humboldt Bay [Yos Sudarso Bay]

#### Collix (Collix) adamata

Prout, 1941

Collix (Collix) adamata Prout 1941

##### Materials

- **Type status:** Holotype. **Occurrence:** sex: f; **Record Level:** ownerInstitutionCode: NHM

##### Distribution

Type locality: SW Celebes [Sulawesi], G. Lampobattang, Parang-bobo Goa, 5000 ft.

#### Collix (Collix) astathes

Prout, 1937

Collix (Collix) astathes Prout 1937

##### Materials

- **Type status:** Syntype. **Occurrence:** sex: 1m, 1f; **Record Level:** ownerInstitutionCode: NHM

##### Distribution

Type locality: Bali (east), Batoeriti, 3500 ft.

#### Collix (Collix) basicristata

Prout, 1923

Collix (Collix) basicristata Prout 1923

##### Materials

- **Type status:** Syntype. **Occurrence:** sex: f; **Record Level:** ownerInstitutionCode: NHM

##### Distribution

Type locality: Lesser Sunda Islands, Flores (south)

#### Collix (Collix) ghosha mayri

Prout, 1941

Collix (Collix) ghosha mayri Prout 1941

##### Materials

- **Type status:** Syntype. **Occurrence:** sex: 3m; **Record Level:** ownerInstitutionCode: NHM

##### Distribution

Type locality: [West Papua] and [Papua New Guinea], Arfak, Mt Siwi, 800 m

##### Notes

The species *C. ghosha* Walker (1862) is illustrated in Holloway (1997)

#### Collix (Collix) rufidorsata rufidorsata

Prout, 1929

Collix (Collix) rufidorsata rufidorsata Prout 1929e

##### Materials

- **Type status:** Holotype. **Occurrence:** sex: m; **Record Level:** ownerInstitutionCode: NHM

##### Distribution

Type locality: Java, Sukabumi, 2000 ft.

##### Notes

The species is illustrated in Holloway (1997)

#### Collix (Collix) stellata oblitera

Prout, 1935

Collix (Collix) stellata oblitera Prout 1935

##### Materials

- **Type status:** Holotype. **Occurrence:** sex: m; **Record Level:** ownerInstitutionCode: NHM

##### Distribution

Type locality: Java (east), Nongkodjadjar

##### Notes

The subspecies *C. stellata oblitera* is described as *C. griseipalpis oblitera*

#### Eupithecia (Eupithecia) excita

Prout, 1958

Eupithecia (Eupithecia) excita Prout 1958

##### Materials

- **Type status:** Holotype. **Occurrence:** sex: m; **Record Level:** ownerInstitutionCode: NHM

##### Distribution

Type locality: Celebes [Sulawesi], Tjamba, near Maros, 1500 ft.

#### Eupithecia (Eupithecia) leucoprora

Prout, 1958

Eupithecia (Eupithecia) leucoprora Prout 1958

##### Materials

- **Type status:** Holotype. **Occurrence:** sex: m; **Record Level:** ownerInstitutionCode: NHM

##### Distribution

Type locality: [West Papua], Mt Goliath, 5000-7000 ft.

#### Eupithecia (Eupithecia) lissopis

Prout, 1958

Eupithecia (Eupithecia) lissopis Prout 1958

##### Materials

- **Type status:** Holotype. **Occurrence:** sex: m; **Record Level:** ownerInstitutionCode: NHM

##### Distribution

Type locality: [West Papua], Mount Goliath, 5000-7000 ft.

#### Glaucoclystis (Gymnoscelis) albicetrata

(Prout 1958)

Glaucoclystis (Gymnoscelis) albicetrata Prout 1958

##### Materials

- **Type status:** Holotype. **Occurrence:** sex: m; **Record Level:** ownerInstitutionCode: NHM

##### Distribution

Type locality: Celebes (west) [Sulawesi], Paloe, G. Tompoe, 2700 ft.

#### Gymnoscelis (Gymnoscelis) anaxia

Prout, 1958

Gymnoscelis (Gymnoscelis) anaxia Prout 1958

##### Materials

- **Type status:** Holotype. **Occurrence:** sex: unknown; **Record Level:** ownerInstitutionCode: NHM

##### Distribution

Type locality: Toekan Bessi Islands [Tukang Besi], Tomia

#### Gymnoscelis (Gymnoscelis) biangulata

Swinhoe, 1902

Gymnoscelis (Gymnoscelis) biangulata Swinhoe 1902

##### Materials

- **Type status:** Syntype. **Occurrence:** sex: f; **Record Level:** ownerInstitutionCode: NHM

##### Distribution

Type locality: Lesser Sunda Islands, Sambawa [Sumbawa]

#### Gymnoscelis (Gymnoscelis) celebensis

Prout, 1958

Gymnoscelis (Gymnoscelis) celebensis Prout 1958

##### Materials

- **Type status:** Holotype. **Occurrence:** sex: unknown; **Record Level:** ownerInstitutionCode: NHM

##### Distribution

Type locality: Celebes (west) [Sulawesi], Paloe, G. Rangkoenau, 900 ft.

##### Notes

The species is described as subspecies of *G. mesophoena celebensis*. The species *G. celebensis* is illustrated in Holloway (1997)

#### Gymnoscelis (Gymnoscelis) derogata griseifusa

Prout, 1958

Gymnoscelis (Gymnoscelis) derogata griseifusa Prout 1958

##### Materials

- **Type status:** Holotype. **Occurrence:** sex: f; **Record Level:** ownerInstitutionCode: NHM

##### Distribution

Type locality: Celebes (west) [Sulawesi], Paloe, G. Tompoe, 2700 ft.

##### Notes

The species *G. derogata* (Walker, 1866) is illustrated in Holloway (1997)

#### Gymnoscelis (Gymnoscelis) festiva buruensis

Prout, 1958

Gymnoscelis (Gymnoscelis) festiva buruensis Prout 1958

##### Materials

- **Type status:** Holotype. **Occurrence:** sex: f; **Record Level:** ownerInstitutionCode: NHM

##### Distribution

Type locality: [Moluccas], Buru, Leksula-Kakal, 2800-3700 ft.

#### Gymnoscelis (Gymnoscelis) festiva jubilata

Prout, 1958

Gymnoscelis (Gymnoscelis) festiva jubilata Prout 1958

##### Materials

- **Type status:** Holotype. **Occurrence:** sex: f; **Record Level:** ownerInstitutionCode: NHM

##### Distribution

Type locality: Celebes [Sulawesi], G. Lampobattang, Parang-bobo Goa, 5000 ft.

#### Gymnoscelis (Gymnoscelis) holoprasia

Prout, 1958

Gymnoscelis (Gymnoscelis) holoprasia Prout 1958

##### Materials

- **Type status:** Holotype. **Occurrence:** sex: m; **Record Level:** ownerInstitutionCode: NHM

##### Distribution

Type locality: Bali (west), Prapetagoeng, 1500 ft.

#### Gymnoscelis (Botys?) imparatalis

(Walker 1866)

Gymnoscelis (Botys?) imparatalis Walker 1866a
Gymnoscelis (Botys?) imparatalis Synonym: *G. semivinosa*

##### Materials

- **Type status:** Syntype. **Occurrence:** sex: m; **Record Level:** ownerInstitutionCode: OUM

##### Distribution

Type locality: Malaysia, Borneo, Sarawak. Type locality of synonym: Java (east)

##### Notes

The species is described from Malaysia, Borneo, Sarawak and deposited in OUM. The synonym G. (Chloroclystis) semivinosa Warren 1896) is described from Java (east) and deposited in NHM

#### Gymnoscelis (Gymnoscelis) nepotalis

Prout, 1958

Gymnoscelis (Gymnoscelis) nepotalis Prout 1958

##### Materials

- **Type status:** Holotype. **Occurrence:** sex: m; **Record Level:** ownerInstitutionCode: NHM

##### Distribution

Type locality: Java (east), Tengger, Singolangoe, 5000 ft.

##### Notes

The species *G. nepotalis* is described as subspecies of *G. latipennis* Prout (1958) and illustrated in Holloway (1997)

#### Gymnoscelis (Gymnoscelis) nigrescens

Warren, 1898

Gymnoscelis (Gymnoscelis) nigrescens Warren 1898

##### Materials

- **Type status:** Holotype. **Occurrence:** sex: f; **Record Level:** ownerInstitutionCode: NHM

##### Distribution

Type locality: [West Papua], Kai Islands, Key [Kai] Islands

#### Gymnoscelis (Gymnoscelis) pallidirufa

Warren, 1897

Gymnoscelis (Gymnoscelis) pallidirufa Warren 1897a

##### Materials

- **Type status:** Holotype. **Occurrence:** sex: f; **Record Level:** ownerInstitutionCode: NHM

##### Distribution

Type locality: Celebes (south) [Sulawesi], Bonthain, 5000-7000 ft.

#### Gymnoscelis (Gymnoscelis) phoenicopus

Prout, 1958

Gymnoscelis (Gymnoscelis) phoenicopus Prout 1958

##### Materials

- **Type status:** Holotype. **Occurrence:** sex: f; **Record Level:** ownerInstitutionCode: NHM

##### Distribution

Type locality: [Moluccas], Ceram (central) [Seram], Manusela, 6000 ft.

##### Notes

The species is illustrated in Holloway (1997)

#### Gymnoscelis (Gymnoscelis) pyrissous

Prout, 1958

Gymnoscelis (Gymnoscelis) pyrissous Prout 1958

##### Materials

- **Type status:** Holotype. **Occurrence:** sex: unknown; **Record Level:** ownerInstitutionCode: NHM

##### Distribution

Type locality: Lesser Sunda Islands, Tambora, low country

##### Notes

The synonym *G. maculilinea* (Warren, 1898) is described from [West Papua], Key [Kai] Islands

#### Mariaba (Mariaba) convoluta

Walker, 1866

Mariaba (Mariaba) convoluta Walker 1866b
Mariaba (Mariaba) convoluta Synonym: *M. ampla*

##### Materials

- **Type status:** Syntype. **Occurrence:** sex: f; **Record Level:** ownerInstitutionCode: OUM

##### Distribution

Type locality: Malaysia, Borneo. Type locality of synonym: Lesser Sunda Islands, Lombok

##### Notes

The species is described from Malaysia, Borneo and deposited in OUM. The synonym *M. (Megatheca?) ampla* (Warren, 1899) is described from Lesser Sunda Islands, Lombok

#### Micrulia (Eupithecia) catocalaria

(Snellen 1881)

Micrulia (Eupithecia) catocalaria Snellen 1881

##### Materials

- **Type status:** Holotype. **Occurrence:** sex: f; **Record Level:** ownerInstitutionCode: NHM

##### Distribution

Type locality: Celebes [Sulawesi], Macassar

##### Notes

The species is illustrated in Holloway (1997)

#### Micrulia (Opistheploce) cinerea

(Warren 1896)

Micrulia (Opistheploce) cinerea Warren 1896

##### Materials

- **Type status:** Holotype. **Occurrence:** sex: m; **Record Level:** ownerInstitutionCode: NHM

##### Distribution

Type locality: [Moluccas], Batchian [Batjan Island]

#### Pasiphila (Chloroclystis) palpata javana

(Prout 1958)

Pasiphila (Chloroclystis) palpata javana Prout 1958

##### Materials

- **Type status:** Holotype. **Occurrence:** sex: m; **Record Level:** ownerInstitutionCode: NHM

##### Distribution

Type locality: Java (east), Mt Moenggal, 9000 ft.

##### Notes

The species *P. palpata* (Walker, 1862) is illustrated in Holloway (1997)

#### Pasiphilodes (Chloroclystis) automola

(Prout 1929)

Pasiphilodes (Chloroclystis) automola Prout 1929b

##### Materials

- **Type status:** Syntype. **Occurrence:** sex: 2f; **Record Level:** ownerInstitutionCode: NHM

##### Distribution

Type locality: [Moluccas], Ceram (central) [Seram], Manusela, 6000 ft.

#### Pasiphilodes (Chloroclystis) diaboeta

(Prout 1958)

Pasiphilodes (Chloroclystis) diaboeta Prout 1958

##### Materials

- **Type status:** Holotype. **Occurrence:** sex: f; **Record Level:** ownerInstitutionCode: NHM

##### Distribution

Type locality: [Moluccas], Seram (central), Manusela, 6000 ft.

#### Pasiphilodes (Chloroclystis) diaschista

(Prout 1958)

Pasiphilodes (Chloroclystis) diaschista Prout 1958

##### Materials

- **Type status:** Holotype. **Occurrence:** sex: m; **Record Level:** ownerInstitutionCode: NHM

##### Distribution

Type locality: [West Papua], Mt Goliath, 5000-7000 ft.

#### Pasiphilodes (Chloroclystis) hypodela

(Prout 1926)

Pasiphilodes (Chloroclystis) hypodela Prout 1926

##### Materials

- **Type status:** Syntype. **Occurrence:** sex: 2m; **Record Level:** ownerInstitutionCode: NHM

##### Distribution

Type locality: [West Papua], Mt Goliath

#### Pasiphilodes (Chloroclystis) isophrica

(Prout 1926)

Pasiphilodes (Chloroclystis) isophrica Prout 1926

##### Materials

- **Type status:** Syntype. **Occurrence:** sex: 1m, 1f; **Record Level:** ownerInstitutionCode: NHM

##### Distribution

Type locality: [West Papua], Mt Goliath, 5000-7000 ft.

#### Pasiphilodes (Rhinoprora) oribates

(Prout 1925)

Pasiphilodes (Rhinoprora) oribates Prout 1925

##### Materials

- **Type status:** Holotype. **Occurrence:** sex: m; **Record Level:** ownerInstitutionCode: NHM

##### Distribution

Type locality: Java, Mt Gedeh

#### Pomasia (Pomasia) euryopis

Meyrick, 1897

Pomasia (Pomasia) euryopis Meyrick 1897

##### Materials

- **Type status:** Holotype. **Occurrence:** sex: m; **Record Level:** ownerInstitutionCode: NHM

##### Distribution

Type locality: nr Kalimantan, Pulo Laut

##### Notes

The species is illustrated in Holloway (1997)

#### Pomasia (Pomasia) gelastis

Meyrick, 1897

Pomasia (Pomasia) gelastis Meyrick 1897

##### Materials

- **Type status:** Holotype. **Occurrence:** sex: m; **Record Level:** ownerInstitutionCode: NHM

##### Distribution

Type locality: nr Kalimantan, Pulo Laut

##### Notes

The species is illustrated in Holloway (1997)

#### Pomasia (Eupithecia) obliterata

(Walker 1866)

Pomasia (Eupithecia) obliterata Walker 1866b
Pomasia (Eupithecia) obliterata Synonym: *P. conferta*

##### Materials

- **Type status:** Syntype. **Occurrence:** sex: f; **Record Level:** ownerInstitutionCode: OUM

##### Distribution

Type locality: Malaysia, Borneo. Type locality of synonym: Kalimantan, Pulo Laut

##### Notes

The species is described from Malaysia, Borneo and deposited in OUM. The synonym P. (Eupithecia) conferta (Swinhoe, 1902) is described from Kalimantan, Pulo Laut. The species *P. obliterata* is illustrated in Holloway (1997)

#### Pomasia (Pomasia) salutaris

Prout, 1929

Pomasia (Pomasia) salutaris Prout 1929c

##### Materials

- **Type status:** Syntype. **Occurrence:** sex: 5m, 2f; **Record Level:** ownerInstitutionCode: NHM

##### Distribution

Type locality: Sumatra, Barisan Range, western slopes, 2500 ft.

##### Notes

The species is illustrated in Holloway (1997)

#### Pseudopolinesia (Pomasia) hebe

(Bethune-Baker 1915)

Pseudopolinesia (Pomasia) hebe Bethune-Baker 1915
Pseudopolinesia (Pomasia) hebe Synonyms: *P. interrupta*, *P. phanoides*, *P. praelustris*

##### Materials

- **Type status:** Syntype. **Occurrence:** sex: m; **Record Level:** ownerInstitutionCode: NHM

##### Distribution

Type locality: British New Guinea [Papua New Guinea], Mt Kebea, 3000 ft. Type localities of synonyms: [West Papua], Oetakwa River, Celebes [Sulawesi], Menado, [Moluccas], Buru, Gamoe, Mrapat, 5000 ft.

##### Notes

The species is described from British New Guinea [Papua New Guinea], Mt Kebea, 3000 ft. Three synonyms *P. interrupta* (Prout 1916) *P. phanoides* (Debauche 1941) and *P. praelustris* (Prout 1933) are described from [West Papua], Oetakwa River, from Celebes [Sulawesi], Menado and from [Moluccas], Buru, Gamoe, Mrapat, 5000 ft.

#### Ptychotheca (Chloroclystis) pallidivirens

(Warren 1903)

Ptychotheca (Chloroclystis) pallidivirens Warren 1903
Ptychotheca (Chloroclystis) pallidivirens Synonym: *P. pallidivirens pullivirens*

##### Materials

- **Type status:** Holotype. **Occurrence:** sex: m; **Record Level:** ownerInstitutionCode: NHM

##### Distribution

Type locality: British New Guinea [Papua New Guinea], Upper Aroa River. Type locality of synonym: Java (east), Nongkodjadjar

##### Notes

The species is described from British New Guinea [Papua New Guinea], Upper Aroa River. The synonym P. (Chloroclystis) pallidivirens pullivirens (Prout, 1935) is described from Java (east), Nongkodjadjar

#### Spiralisigna (Gymnoscelis) minutissima

(Swinhoe 1902)

Spiralisigna (Gymnoscelis) minutissima Swinhoe 1902

##### Materials

- **Type status:** Syntype. **Occurrence:** sex: m, f; **Record Level:** ownerInstitutionCode: NHM

##### Distribution

Type locality: Lesser Sunda Islands, Sambawa [Sumbawa]

##### Notes

The species is illustrated in Holloway (1997)

#### Syncosmia (Chloroclystis) craspedozona craspedozona

(Prout 1958)

Syncosmia (Chloroclystis) craspedozona craspedozona Prout 1958

##### Materials

- **Type status:** Holotype. **Occurrence:** sex: f; **Record Level:** ownerInstitutionCode: NHM

##### Distribution

Type locality: Bali (east), Batoeriti, 3500 ft.

#### Syncosmia (Chloroclystis) craspedozona heanis

(Prout 1958)

Syncosmia (Chloroclystis) craspedozona heanis Prout 1958

##### Materials

- **Type status:** Holotype. **Occurrence:** sex: f; **Record Level:** ownerInstitutionCode: NHM

##### Distribution

Type locality: [Moluccas], Seram (central), Manusela, 6000 ft.

#### Syncosmia (Chloroclystis) dissographa

(Prout 1958)

Syncosmia (Chloroclystis) dissographa Prout 1958

##### Materials

- **Type status:** Holotype. **Occurrence:** sex: unknown; **Record Level:** ownerInstitutionCode: NHM

##### Distribution

Type locality: Celebes [Sulawesi], G. Lampobattang, Parang-bobo Goa, 5000 ft.

##### Notes

The species is illustrated in Holloway (1997)

#### Syncosmia (Chloroclystis) eugerys

(Prout 1929)

Syncosmia (Chloroclystis) eugerys Prout 1929b

##### Materials

- **Type status:** Syntype. **Occurrence:** sex: 3m, 2f; **Record Level:** ownerInstitutionCode: NHM

##### Distribution

Type locality: [Moluccas], Ceram [Seram] (central), Manusela, 6000 ft.

#### Tripteridia (Micromia) conquadrata

(Prout 1958)

Tripteridia (Micromia) conquadrata Prout 1958

##### Materials

- **Type status:** Holotype. **Occurrence:** sex: m; **Record Level:** ownerInstitutionCode: NHM

##### Distribution

Type locality: [West Papua], Mt Goliath, 5000-7000 ft.

#### Tripteridia (Micromia) dilopha

(Prout 1958)

Tripteridia (Micromia) dilopha Prout 1958

##### Materials

- **Type status:** Holotype. **Occurrence:** sex: m; **Record Level:** ownerInstitutionCode: NHM

##### Distribution

Type locality: [West Papua], Mt Goliath, 5000-7000 ft.

#### Tripteridia (Micromia) dympna

(Prout 1958)

Tripteridia (Micromia) dympna Prout 1958

##### Materials

- **Type status:** Holotype. **Occurrence:** sex: m; **Record Level:** ownerInstitutionCode: NHM

##### Distribution

Type locality: [West Papua], Mt Goliath, 5000-7000 ft.

#### Tripteridia (Micromia) ectocosma

(Prout 1958)

Tripteridia (Micromia) ectocosma Prout 1958

##### Materials

- **Type status:** Holotype. **Occurrence:** sex: m; **Record Level:** ownerInstitutionCode: NHM

##### Distribution

Type locality: [West Papua], Mt Goliath, 5000-7000 ft., ca 139° longit.

#### Tripteridia (Prosthetopteryx) eusemozona

(Prout 1916)

Tripteridia (Prosthetopteryx) eusemozona Prout 1916

##### Materials

- **Type status:** Holotype. **Occurrence:** sex: m; **Record Level:** ownerInstitutionCode: NHM

##### Distribution

Type locality: [West Papua], Mt Goliath, 5000-7000 ft.

#### Tripteridia (Micromia) euthynsis

(Prout 1958)

Tripteridia (Micromia) euthynsis Prout 1958

##### Materials

- **Type status:** Holotype. **Occurrence:** sex: m; **Record Level:** ownerInstitutionCode: NHM

##### Distribution

Type locality: [West Papua], Mt Goliath, 5000-7000 ft.

#### Tripteridia (Micromia) monochasma

(Prout 1958)

Tripteridia (Micromia) monochasma Prout 1958

##### Materials

- **Type status:** Holotype. **Occurrence:** sex: m; **Record Level:** ownerInstitutionCode: NHM

##### Distribution

Type locality: [West Papua], Mt Goliath, 5000-7000 ft.

#### Tripteridia (Micromia) ni

(Prout 1958)

Tripteridia (Micromia) ni Prout 1958

##### Materials

- **Type status:** Holotype. **Occurrence:** sex: m; **Record Level:** ownerInstitutionCode: NHM

##### Distribution

Type locality: [West Papua], Mt Goliath, 5000-7000 ft.

#### Tripteridia (Micromia) novenaria

(Prout 1958)

Tripteridia (Micromia) novenaria Prout 1958

##### Materials

- **Type status:** Holotype. **Occurrence:** sex: m; **Record Level:** ownerInstitutionCode: NHM

##### Distribution

Type locality: [West Papua], Mt Goliath, 5000-7000 ft.

#### Tripteridia (Micromia) scotochlaena

(Prout 1931)

Tripteridia (Micromia) scotochlaena Prout 1931

##### Materials

- **Type status:** Syntype. **Occurrence:** sex: 2m; **Record Level:** ownerInstitutionCode: NHM

##### Distribution

Type locality: [West Papua], Mt Goliath, 5000-7000 ft.

#### Tripteridia (Eupithecia) synclinogramma

(Prout 1916)

Tripteridia (Eupithecia) synclinogramma Prout 1916

##### Materials

- **Type status:** Holotype. **Occurrence:** sex: m; **Record Level:** ownerInstitutionCode: NHM

##### Distribution

Type locality: [West Papua], Mt Goliath, 5000-7000 ft.

#### Tripteridia (Micromia) thaumasia

(Prout 1958)

Tripteridia (Micromia) thaumasia Prout 1958

##### Materials

- **Type status:** Holotype. **Occurrence:** sex: m; **Record Level:** ownerInstitutionCode: NHM

##### Distribution

Type locality: [West Papua], Mt Goliath, 5000-7000 ft.

#### Ziridava (Ziridava) baliensis

Prout, 1958

Ziridava (Ziridava) baliensis Prout 1958

##### Materials

- **Type status:** Holotype. **Occurrence:** sex: unknown; **Record Level:** ownerInstitutionCode: NHM

##### Distribution

Type locality: Bali, Batoeriti, 3500 ft.

##### Notes

The species *Z. baliensis* Prout (1958) is described as subspecies of *Z. xylinaria* Walker (1863). The synonym *Z. xylinaria florensis* Prout (1958) is described from Lesser Sunda Islands, Flores (south)

#### Ziridava (Ziridava) xylinaria

Walker, 1863

Ziridava (Ziridava) xylinaria Walker 1863
Ziridava (Ziridava) xylinaria Synonyms: *Z. xylinaria subaequata*, *Z. subrubida*

##### Materials

- **Type status:** Syntype. **Occurrence:** sex: f; **Record Level:** ownerInstitutionCode: OUM

##### Distribution

Type locality: Malaysia, Borneo, Sarawak. Type locality of synonym: [Moluccas], Ceram [Seram] (central), Manusela and Celebes (south) [Sulawesi], Bonthian, Indrulaman, 2300 ft.

##### Notes

The species is described from Malaysia, Borneo, Sarawak, deposited in OUM and illustrated in Holloway (1997). Two synonyms *Z. xylinaria subaequata* Prout (1929) and *Z. subrubida* Warren (1897) are described from [Moluccas], Ceram [Seram] (central), Manusela and Celebes (south) [Sulawesi], Bonthian, Indrulaman, 2300 ft.

#### Larentiini

#### Photoscotosia (Photoscotosia) multiplicata

Warren, 1898

Photoscotosia (Photoscotosia) multiplicata Warren 1898

##### Materials

- **Type status:** Syntype. **Occurrence:** sex: 2f; **Record Level:** ownerInstitutionCode: NHM

##### Distribution

Type locality: Java, Mt Arjuno

##### Notes

The synonym *P. multiplicata erebenna* Prout (1935) is described from Java (east), Mt Moenggal, 9000 ft. and Bromo to Caldeira

#### Melanthiini

#### Horisme (Collix) boarmiata boarmiata

(Snellen 1881)

Horisme (Collix) boarmiata boarmiata Snellen 1881

##### Materials

- **Type status:** Syntype. **Occurrence:** sex: 1m, 1f; **Record Level:** ownerInstitutionCode: NBC

##### Distribution

Type locality: Celebes [Sulawesi], Lokka

#### Horisme (Horisme) boarmiata grandescens

Prout, 1929

Horisme (Horisme) boarmiata grandescens Prout 1929a

##### Materials

- **Type status:** Holotype. **Occurrence:** sex: f; **Record Level:** ownerInstitutionCode: NHM

##### Distribution

Type locality: [Moluccas], Buru, Rana

#### Horisme (Horisme) boarmiata inconstans

Prout, 1941

Horisme (Horisme) boarmiata inconstans Prout 1941

##### Materials

- **Type status:** Syntype. **Occurrence:** sex: many, m, f; **Record Level:** ownerInstitutionCode: NHM

##### Distribution

Type locality: Java (east), Bali, Singolangoe, Tengger, 1500 m

#### Horisme (Horisme) boarmiata serangica

Prout, 1941

Horisme (Horisme) boarmiata serangica Prout 1941

##### Materials

- **Type status:** Syntype. **Occurrence:** sex: 1m, 1f; **Record Level:** ownerInstitutionCode: NHM

##### Distribution

Type locality: [Moluccas], Seram (central), Manusela, 6000 ft.

#### Horisme (Horisme) brooksi

Prout, 1941

Horisme (Horisme) brooksi Prout 1941

##### Materials

- **Type status:** Holotype. **Occurrence:** sex: m; **Record Level:** ownerInstitutionCode: NHM

##### Distribution

Type locality: Sumatra, Dempo, 4000 ft.

#### Horisme (Horisme) dealbata

Inoue, 1992

Horisme (Horisme) dealbata Inoue 1992

##### Materials

- **Type status:** Holotype. **Occurrence:** sex: m; **Record Level:** ownerInstitutionCode: NHM

##### Distribution

Type locality: Celebes (south) [Sulawesi], near N border, Puncak Dingin, 1700 m

#### Horisme (Horisme) invicta

Prout, 1941

Horisme (Horisme) invicta Prout 1941

##### Materials

- **Type status:** Syntype. **Occurrence:** sex: 2f; **Record Level:** ownerInstitutionCode: NHM

##### Distribution

Type locality: Sumatra (west), Korinchi, 7300 ft.

#### Horisme (Horisme) praemaculata

Prout, 1929

Horisme (Horisme) praemaculata Prout 1929b

##### Materials

- **Type status:** Holotype. **Occurrence:** sex: m; **Record Level:** ownerInstitutionCode: NHM

##### Distribution

Type locality: [Moluccas], Buru, Fakal

#### Horisme (Horisme) semirufata goliathi

Prout, 1941

Horisme (Horisme) semirufata goliathi Prout 1941

##### Materials

- **Type status:** Syntype. **Occurrence:** sex: 1m, 2f; **Record Level:** ownerInstitutionCode: NHM

##### Distribution

Type locality: [West Papua], Mt Goliath, 5000-7000 ft.

#### Horisme (Horisme) steretica

Prout, 1941

Horisme (Horisme) steretica Prout 1941

##### Materials

- **Type status:** Syntype. **Occurrence:** sex: 5m; **Record Level:** ownerInstitutionCode: NHM

##### Distribution

Type locality: [West Papua], Mt Goliath, 5000-7000 ft.

##### Notes

Scoble (1999) “holotype male”, however, in the original description five specimens are mentioned indirectly

#### Trichopterygini

#### Brabira (Brabira) mesoschides

Prout, 1929

Brabira (Brabira) mesoschides Prout 1929d

##### Materials

- **Type status:** Syntype. **Occurrence:** sex: f; **Record Level:** ownerInstitutionCode: NHM

##### Distribution

Type locality: [West Papua], Weyland Mountains, Mt Kunupi, 6000 ft.

#### Carige (Carige) bicuspis

Prout, 1931

Carige (Carige) bicuspis Prout 1931

##### Materials

- **Type status:** Syntype. **Occurrence:** sex: 1m,1f; **Record Level:** ownerInstitutionCode: NHM

##### Distribution

Type locality: Sumatra (west), Sungei Kumbang, Korintji district, 4500 ft.

#### Carige (Carige) combinata

Warren, 1899

Carige (Carige) combinata Warren 1899a

##### Materials

- **Type status:** Holotype. **Occurrence:** sex: m; **Record Level:** ownerInstitutionCode: NHM

##### Distribution

Type locality: Lesser Sunda Islands, Flores (south)

#### Dystypoptila (Dystypoptila) hebes

Prout, 1958

Dystypoptila (Dystypoptila) hebes Prout 1958

##### Materials

- **Type status:** Holotype. **Occurrence:** sex: m; **Record Level:** ownerInstitutionCode: NHM

##### Distribution

Type locality: Celebes (west) [Sulawesi], Paloe, G. Rangkoenau, 1800 ft.

#### Dystypoptila (Dystypoptila) triangularis

Warren, 1895

Dystypoptila (Dystypoptila) triangularis Warren 1895

##### Materials

- **Type status:** Holotype. **Occurrence:** sex: m; **Record Level:** ownerInstitutionCode: NHM

##### Distribution

Type locality: Sumatra, Padang

##### Notes

The species is illustrated in Holloway (1997)

#### Episteira (Episteira) colligata

Warren, 1899

Episteira (Episteira) colligata Warren 1899a

##### Materials

- **Type status:** Holotype. **Occurrence:** sex: m; **Record Level:** ownerInstitutionCode: NHM

##### Distribution

Type locality: Lesser Sunda Islands, Flores (south)

#### Episteira (Episteira) delicata isoepes

Prout, 1958

Episteira (Episteira) delicata isoepes Prout 1958

##### Materials

- **Type status:** Holotype. **Occurrence:** sex: unknown; **Record Level:** ownerInstitutionCode: NHM

##### Distribution

Type locality: [Moluccas], Ceram [Seram] (central), Manusela

#### Episteira (Sauris) infirma

(Swinhoe 1902)

Episteira (Sauris) infirma Swinhoe 1902

##### Materials

- **Type status:** Syntype. **Occurrence:** sex: f; **Record Level:** ownerInstitutionCode: NHM

##### Distribution

Type locality: nr Kalimantan, Pulo Laut

##### Notes

The species is illustrated in Holloway (1997)

#### Episteira (Sauris) nigrilinearia euneta

(Prout 1958)

Episteira (Sauris) nigrilinearia euneta Prout 1958

##### Materials

- **Type status:** Holotype. **Occurrence:** sex: f; **Record Level:** ownerInstitutionCode: NHM

##### Distribution

Type locality: Celebes (west) [Sulawesi], Paloe, Loda, 4000 ft.

#### Goniopteroloba (Goniopteroloba) carigodes

Prout, 1931

Goniopteroloba (Goniopteroloba) carigodes Prout 1931

##### Materials

- **Type status:** Holotype. **Occurrence:** sex: m; **Record Level:** ownerInstitutionCode: NHM

##### Distribution

Type locality: Sumatra (west), Sungei Kumbang, 4500 ft.

#### Goniopteroloba (Goniopteroloba) pallida pallida

Warren, 1902

Goniopteroloba (Goniopteroloba) pallida pallida Warren 1902

##### Materials

- **Type status:** Syntype. **Occurrence:** sex: 2m; **Record Level:** ownerInstitutionCode: NHM

##### Distribution

Type locality: Celebes [Sulawesi], Bonthain, 3000-7000 ft.

#### Goniopteroloba (Goniopteroloba) pallida pangeanensis

Prout, 1958

Goniopteroloba (Goniopteroloba) pallida pangeanensis Prout 1958

##### Materials

- **Type status:** Holotype. **Occurrence:** sex: m; **Record Level:** ownerInstitutionCode: NHM

##### Distribution

Type locality: Celebes (south-west) [Sulawesi], Pangean, near Maros, 2000 ft.

#### Hypocometa (Sauris) definita

(Joicey & Talbot 1917)

Hypocometa (Sauris) definita Joicey and Talbot 1917
Hypocometa (Sauris) definita Synonym: *H. hypelaina*

##### Materials

- **Type status:** Holotype. **Occurrence:** sex: f; **Record Level:** ownerInstitutionCode: NHM

##### Distribution

Type locality: [West Papua], Arfak Mts, Angi Lakes, 6000 ft. Type locality of synonym: [Moluccas], Ceram [Seram] (central), Manusela

##### Notes

The species is described from [West Papua], Arfak Mts, Angi Lakes, 6000 ft. The synonym H. (Phthonoloba) hypelaina (Prout, 1929) is described from [Moluccas], Ceram [Seram] (central), Manusela

#### Hypocometa (Phthonoloba) praeeminens

(Prout 1916)

Hypocometa (Phthonoloba) praeeminens Prout 1916

##### Materials

- **Type status:** Holotype. **Occurrence:** sex: unknown; **Record Level:** ownerInstitutionCode: NHM

##### Distribution

Type locality: [West Papua], Mt Goliath, 5000-7000 ft.

#### Hypocometa (Hypocometa) rufulata

Warren, 1899

Hypocometa (Hypocometa) rufulata Warren 1899b

##### Materials

- **Type status:** Syntype. **Occurrence:** sex: 2f; **Record Level:** ownerInstitutionCode: NHM

##### Distribution

Type locality: Lesser Sunda Islands, Flores (south)

#### Phthonoloba (Steirophora) acrolophites

(Prout 1926)

Phthonoloba (Steirophora) acrolophites Prout 1926

##### Materials

- **Type status:** Holotype. **Occurrence:** sex: m; **Record Level:** ownerInstitutionCode: NHM

##### Distribution

Type locality: Java, Mt Gedeh, 7500 ft.

#### Phthonoloba (Steirophora) altitudinum

(Prout 1931)

Phthonoloba (Steirophora) altitudinum Prout 1931

##### Materials

- **Type status:** Holotype. **Occurrence:** sex: m; **Record Level:** ownerInstitutionCode: NHM

##### Distribution

Type locality: Sumatra (west), Korintji, 7300 ft.

#### Phthonoloba (Steirophora) auratisquama

(Warren 1897)

Phthonoloba (Steirophora) auratisquama Warren 1897a

##### Materials

- **Type status:** Syntype. **Occurrence:** sex: 1m, 6f; **Record Level:** ownerInstitutionCode: NHM

##### Distribution

Type locality: Java (west), (south), Bandong, South Java, West Java

#### Phthonoloba (Synneurodes) brevipalpis

(Warren 1899)

Phthonoloba (Synneurodes) brevipalpis Warren 1899a

##### Materials

- **Type status:** Holotype. **Occurrence:** sex: m; **Record Level:** ownerInstitutionCode: NHM

##### Distribution

Type locality: Lesser Sunda Islands, Flores (south)

#### Phthonoloba (Sauris) graphica

(Prout 1916)

Phthonoloba (Sauris) graphica Prout 1916

##### Materials

- **Type status:** Holotype. **Occurrence:** sex: unknown; **Record Level:** ownerInstitutionCode: NHM

##### Distribution

Type locality: [West Papua], Mt Goliath, 5000-7000 ft.

#### Phthonoloba (Steirophora) micans

(Prout 1929)

Phthonoloba (Steirophora) micans Prout 1929b

##### Materials

- **Type status:** Syntype. **Occurrence:** sex: 4m, 2f; **Record Level:** ownerInstitutionCode: NHM

##### Distribution

Type locality: [Moluccas], Seram (central), Manusela

#### Phthonoloba (Steirophora) punctatissima

(Warren 1897)

Phthonoloba (Steirophora) punctatissima Warren 1897a

##### Materials

- **Type status:** Holotype. **Occurrence:** sex: m; **Record Level:** ownerInstitutionCode: NHM

##### Distribution

Type locality: Celebes [Sulawesi], Bonthain, 3000-7000 ft.

#### Sauris (Remodes?) angulosa

(Warren 1896)

Sauris (Remodes?) angulosa Warren 1896

##### Materials

- **Type status:** Syntype. **Occurrence:** sex: 2f; **Record Level:** ownerInstitutionCode: NHM

##### Distribution

Type locality: [Moluccas], Amboina [nr Seram, Ambon]

#### Sauris (Sauris) basilia

Prout, 1958

Sauris (Sauris) basilia Prout 1958

##### Materials

- **Type status:** Holotype. **Occurrence:** sex: f; **Record Level:** ownerInstitutionCode: NHM

##### Distribution

Type locality: Celebes [Sulawesi], Gunong Lampobattang, Parang-bobo Goa, 5000 ft.

#### Sauris (Sauris) buruensis

Prout, 1929

Sauris (Sauris) buruensis Prout 1929b

##### Materials

- **Type status:** Holotype. **Occurrence:** sex: m; **Record Level:** ownerInstitutionCode: NHM

##### Distribution

Type locality: [Moluccas], Buru, Gamoe Mrapat, 5000 ft.

#### Sauris (Remodes) eupitheciata

(Snellen 1881)

Sauris (Remodes) eupitheciata Snellen 1881

##### Materials

- **Type status:** Syntype. **Occurrence:** sex: 3f; **Record Level:** ownerInstitutionCode: NBC

##### Distribution

Type locality: Celebes [Sulawesi], Macassar

##### Notes

The species is illustrated in Holloway (1997)

#### Sauris (Phthonoloba) imbecilla

(Swinhoe 1902)

Sauris (Phthonoloba) imbecilla Swinhoe 1902

##### Materials

- **Type status:** Syntype. **Occurrence:** sex: m; **Record Level:** ownerInstitutionCode: NHM

##### Distribution

Type locality: [West Papua], Kapaur

#### Sauris (Sauris) muscosa muscosa

Rothschild, 1916

Sauris (Sauris) muscosa muscosa Rothschild 1916

##### Materials

- **Type status:** Holotype. **Occurrence:** sex: f; **Record Level:** ownerInstitutionCode: NHM

##### Distribution

Type locality: [West Papua], Snow Mountains, Utakwa [Oetakwa] River, 3000 ft.

#### Sauris (Sauris) muscosa pleonectes

Prout, 1958

Sauris (Sauris) muscosa pleonectes Prout 1958

##### Materials

- **Type status:** Holotype. **Occurrence:** sex: f; **Record Level:** ownerInstitutionCode: NHM

##### Distribution

Type locality: Celebes (west) [Sulawesi], Paloe, G. Rangkoenau, 1800 ft.

#### Sauris (Sauris) oetakwana

Prout, 1958

Sauris (Sauris) oetakwana Prout 1958

##### Materials

- **Type status:** Holotype. **Occurrence:** sex: unknown; **Record Level:** ownerInstitutionCode: NHM

##### Distribution

Type locality: [West Papua], Snow Mts, near Oetakwa River, up to 3500 ft.

#### Sauris (Sauris) othnia

Prout, 1958

Sauris (Sauris) othnia Prout 1958

##### Materials

- **Type status:** Holotype. **Occurrence:** sex: m; **Record Level:** ownerInstitutionCode: NHM

##### Distribution

Type locality: [Moluccas], Batchian [Batjan Island]

#### Sauris (Pseudoschista) pallidipalpis

(Prout 1916)

Sauris (Pseudoschista) pallidipalpis Prout 1916

##### Materials

- **Type status:** Holotype. **Occurrence:** sex: m; **Record Level:** ownerInstitutionCode: NHM

##### Distribution

Type locality: [West Papua], Lower Oetakwa River

#### Sauris (Remodes) pallidiplaga

(Warren 1897)

Sauris (Remodes) pallidiplaga Warren 1897b

##### Materials

- **Type status:** Holotype. **Occurrence:** sex: m; **Record Level:** ownerInstitutionCode: NHM

##### Distribution

Type locality: Java (west), Mt Gede, 4000 ft.

##### Notes

The species is illustrated in Holloway (1997)

#### Sauris (Sauris) preptochaetes

Prout, 1929

Sauris (Sauris) preptochaetes Prout 1929b

##### Materials

- **Type status:** Syntype. **Occurrence:** sex: 5m, 5f; **Record Level:** ownerInstitutionCode: NHM

##### Distribution

Type locality: [Moluccas], Seram (central), Manusela

#### Sauris (Remodes?) rubriplaga

(Warren 1899)

Sauris (Remodes?) rubriplaga Warren 1899a

##### Materials

- **Type status:** Holotype. **Occurrence:** sex: f; **Record Level:** ownerInstitutionCode: NHM

##### Distribution

Type locality: [Moluccas], Obi, Laiwui

#### Sauris (Helminthoceras) sinuaticornis

(Warren 1896)

Sauris (Helminthoceras) sinuaticornis Warren 1896

##### Materials

- **Type status:** Holotype. **Occurrence:** sex: f; **Record Level:** ownerInstitutionCode: NHM

##### Distribution

Type locality: [West Papua], Humboldt Bay [Yos Sudarso Bay]

#### Sauris (Coptogonia) turpipennis

(Warren 1896)

Sauris (Coptogonia) turpipennis Warren 1896

##### Materials

- **Type status:** Holotype. **Occurrence:** sex: m; **Record Level:** ownerInstitutionCode: NHM

##### Distribution

Type locality: [Moluccas], Batchian [Batjan Island]

#### Sauris (Sauris) usta asema

Prout, 1958

Sauris (Sauris) usta asema Prout 1958

##### Materials

- **Type status:** Holotype. **Occurrence:** sex: m; **Record Level:** ownerInstitutionCode: NHM

##### Distribution

Type locality: Java (east), Nongkodjadjar, 4000 ft.

##### Notes

The species *Sauris usta* (Warren, 1895) is illustrated in Holloway (1997)

#### Tympanota (Megaloba) admeta

(Prout 1958)

Tympanota (Megaloba) admeta Prout 1958

##### Materials

- **Type status:** Holotype. **Occurrence:** sex: f; **Record Level:** ownerInstitutionCode: NHM

##### Distribution

Type locality: [Moluccas], Seram (central), Manusela

#### Tympanota (Sauris) arfakensis arfakensis

(Joicey & Talbot 1917)

Tympanota (Sauris) arfakensis arfakensis Joicey and Talbot 1917

##### Materials

- **Type status:** Holotype. **Occurrence:** sex: f; **Record Level:** ownerInstitutionCode: NHM

##### Distribution

Type locality: [West Papua], Arfak Mts, Angi Lakes, 6000 ft.

##### Notes

The species is illustrated in Holloway (1997)

#### Tympanota (Sauris) arfakensis catopercna

(Prout 1958)

Tympanota (Sauris) arfakensis catopercna Prout 1958

##### Materials

- **Type status:** Holotype. **Occurrence:** sex: unknown; **Record Level:** ownerInstitutionCode: NHM

##### Distribution

Type locality: [Moluccas], Buru (central), Mrapat, 5000 ft.

#### Tympanota (Sauris) ceramica

(Rothschild 1915)

Tympanota (Sauris) ceramica Rothschild 1915

##### Materials

- **Type status:** Holotype. **Occurrence:** sex: f; **Record Level:** ownerInstitutionCode: NHM

##### Distribution

Type locality: [Moluccas], Seram (central), Manusela, 650 m

##### Notes

The species is illustrated in Holloway (1997)

#### Tympanota (Megaloba) crypsipyrrha

(Prout 1916)

Tympanota (Megaloba) crypsipyrrha Prout 1916

##### Materials

- **Type status:** Holotype. **Occurrence:** sex: unknown; **Record Level:** ownerInstitutionCode: NHM

##### Distribution

Type locality: [West Papua], Mt Goliath

#### Tympanota (Sauris) erecta sententiosa

(Prout 1958)

Tympanota (Sauris) erecta sententiosa Prout 1958

##### Materials

- **Type status:** Holotype. **Occurrence:** sex: f; **Record Level:** ownerInstitutionCode: NHM

##### Distribution

Type locality: [Moluccas], Seram (central), Manusela, 6000 ft.

##### Notes

The species T. (Sauris) erecta Warren (1895) is illustrated in Holloway (1997)

#### Tympanota (Megaloba) loxobasma

(Prout 1958)

Tympanota (Megaloba) loxobasma Prout 1958

##### Materials

- **Type status:** Holotype. **Occurrence:** sex: f; **Record Level:** ownerInstitutionCode: NHM

##### Distribution

Type locality: [West Papua], Mt Goliath, 5000-7000 ft.

#### Tympanota (Sauris) olearia

(Prout 1958)

Tympanota (Sauris) olearia Prout 1958

##### Materials

- **Type status:** Holotype. **Occurrence:** sex: m; **Record Level:** ownerInstitutionCode: NHM

##### Distribution

Type locality: SW Celebes [Sulawesi], G. Lampobattang, Parang-bobo Goa, 5000 ft.

#### Tympanota (Megaloba) postrubidaria

(Rothschild 1916)

Tympanota (Megaloba) postrubidaria Rothschild 1916

##### Materials

- **Type status:** Syntype. **Occurrence:** sex: 2f; **Record Level:** ownerInstitutionCode: NHM

##### Distribution

Type locality: [West Papua], Snow Mountains, Utakwa [Oetakwa] River, 2500-3000 ft.

#### Tympanota (Sauris) ptychosyrma

(Prout 1958)

Tympanota (Sauris) ptychosyrma Prout 1958

##### Materials

- **Type status:** Holotype. **Occurrence:** sex: m; **Record Level:** ownerInstitutionCode: NHM

##### Distribution

Type locality: Celebes (west) [Sulawesi], Paloe, G. Tompoe, 2700 ft.

#### Xanthorhoini

#### Gonanticlea (Gonanticlea) albizona

Prout, 1928

Gonanticlea (Gonanticlea) albizona Prout 1928

##### Materials

- **Type status:** Syntype. **Occurrence:** sex: m, f; **Record Level:** ownerInstitutionCode: NHM

##### Distribution

Type locality: Sumatra, Slopes of Mt Korintji, 7300 ft.

#### Gonanticlea (Cidaria) euclidiata

(Snellen 1881)

Gonanticlea (Cidaria) euclidiata Snellen 1881

##### Materials

- **Type status:** Syntype. **Occurrence:** sex: 2f; **Record Level:** ownerInstitutionCode: NHRS

##### Distribution

Type locality: Celebes [Sulawesi], Lokka

#### Gonanticlea (Gonanticlea) multistriata

Warren, 1896

Gonanticlea (Gonanticlea) multistriata Warren 1896

##### Materials

- **Type status:** Holotype. **Occurrence:** sex: f; **Record Level:** ownerInstitutionCode: NHM

##### Distribution

Type locality: Java (west), Java (west)

#### Gonanticlea (Gonanticlea) penicilla penicilla

Prout, 1932

Gonanticlea (Gonanticlea) penicilla penicilla Prout 1932b

##### Materials

- **Type status:** Holotype. **Occurrence:** sex: m; **Record Level:** ownerInstitutionCode: NHM

##### Distribution

Type locality: Sumatra (west), Korintji, 4500 ft.

#### Gonanticlea (Gonanticlea) penicilla amblia

Prout, 1935

Gonanticlea (Gonanticlea) penicilla amblia Prout 1935

##### Materials

- **Type status:** Syntype. **Occurrence:** sex: 3m, 1f; **Record Level:** ownerInstitutionCode: NHM

##### Distribution

Type locality: Java (east), Nongkodjadjar, Singolangoe

#### Gonanticlea (Gonanticlea) siphla

Prout, 1939

Gonanticlea (Gonanticlea) siphla Prout 1939

##### Materials

- **Type status:** Holotype. **Occurrence:** sex: m; **Record Level:** ownerInstitutionCode: NHM

##### Distribution

Type locality: Celebes [Sulawesi], Paloe, Gunong Rangkoenau, 1800 ft.

#### Gonanticlea (Gonanticlea) sublustris sublustris

Warren, 1903

Gonanticlea (Gonanticlea) sublustris sublustris Warren 1903
Gonanticlea (Gonanticlea) sublustris sublustris Synonym: *G. subpilosa*

##### Materials

- **Type status:** Holotype. **Occurrence:** sex: m; **Record Level:** ownerInstitutionCode: NHM

##### Distribution

Type locality: British New Guinea [Papua New Guinea], Upper Aroa River. Type locality of synonym: [Moluccas], Batjan

##### Notes

The species is described from British New Guinea [Papua New Guinea], Upper Aroa River. The synonym *G. subpilosa* Warren (1904) is described from [Moluccas], Batjan

#### Gonanticlea (Gonanticlea) sublustris stagnatilis

Prout, 1939

Gonanticlea (Gonanticlea) sublustris stagnatilis Prout 1939

##### Materials

- **Type status:** Syntype. **Occurrence:** sex: 7m, 1f; **Record Level:** ownerInstitutionCode: NHM

##### Distribution

Type locality: [Moluccas], Ceram [Seram] (central), Manusela, 6000 ft. and 3000 ft.

#### Loxofidonia (Loxofidonia) bareconia pallidistriga

Prout, 1937

Loxofidonia (Loxofidonia) bareconia pallidistriga Prout 1937

##### Materials

- **Type status:** Syntype. **Occurrence:** sex: 4m, 1f; **Record Level:** ownerInstitutionCode: NHM

##### Distribution

Type locality: Bali (east), Batoeriti, 3500 ft.

#### Loxofidonia (Loxofidonia) hexasticha

Prout, 1941

Loxofidonia (Loxofidonia) hexasticha Prout 1941

##### Materials

- **Type status:** Holotype. **Occurrence:** sex: f; **Record Level:** ownerInstitutionCode: NHM

##### Distribution

Type locality: Celebes (south-west) [Sulawesi], Tjamba, near Maros, 1500 ft.

#### Loxofidonia (Loxofidonia) sigmata sigmata

Prout, 1941

Loxofidonia (Loxofidonia) sigmata sigmata Prout 1941

##### Materials

- **Type status:** Holotype. **Occurrence:** sex: m; **Record Level:** ownerInstitutionCode: NHM

##### Distribution

Type locality: Celebes (west) [Sulawesi], Gunong Tompoe, 2700 ft.

#### Loxofidonia (Loxofidonia) sigmata lipernes

Prout, 1941

Loxofidonia (Loxofidonia) sigmata lipernes Prout 1941

##### Materials

- **Type status:** Holotype. **Occurrence:** sex: m; **Record Level:** ownerInstitutionCode: NHM

##### Distribution

Type locality: [West Papua], Fak Fak, 1700 ft.

#### Scotocyma (Scotocyma) sumatrensis

Schmidt, 2005

Scotocyma (Scotocyma) sumatrensis Schmidt 2005

##### Materials

- **Type status:** Holotype. **Occurrence:** sex: m; **Record Level:** ownerInstitutionCode: ZSM, M. Sommerer coll.

##### Distribution

Type locality: Sumatra, E of Lake Toba, ‘Holzweg 4’, 1150 m

##### Notes

**Figure 1.**
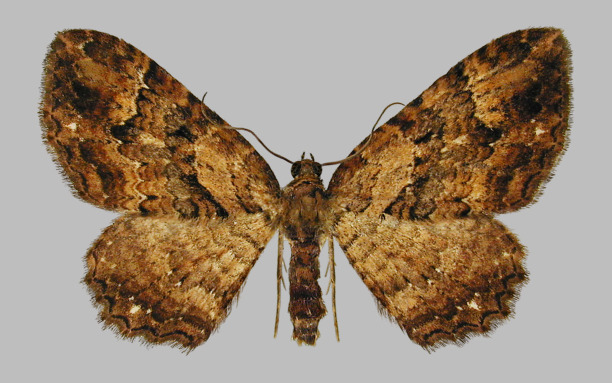
*Scotocyma sumatrensis* Schmidt, 2005. Adult, male, above.

Fig. 1. The species is illustrated in Schmidt (2005), Schmidt (2006b)

#### Xanthorhoe (Xanthorhoe) callisthenes

Prout, 1922

Xanthorhoe (Xanthorhoe) callisthenes Prout 1922b

##### Materials

- **Type status:** Syntype. **Occurrence:** sex: 4m, 4f; **Record Level:** ownerInstitutionCode: NHM

##### Distribution

Type locality: [Moluccas], Ceram [Seram] (central), Manusela, 6000 ft.

#### Xanthorhoe (Xanthorhoe) everetti

Warren, 1897

Xanthorhoe (Xanthorhoe) everetti Warren 1897a

##### Materials

- **Type status:** Syntype. **Occurrence:** sex: 2m, 1f; **Record Level:** ownerInstitutionCode: NHM

##### Distribution

Type locality: Celebes (south) [Sulawesi], Bonthian, 5000-7000 ft.

##### Notes

The synonym *X. roseopicta* Warren (1903) is described from Celebes [Sulawesi]

#### Xanthorhoe (Xanthorhoe) fissiferula

Prout, 1939

Xanthorhoe (Xanthorhoe) fissiferula Prout 1939

##### Materials

- **Type status:** Holotype. **Occurrence:** sex: m; **Record Level:** ownerInstitutionCode: NHM

##### Distribution

Type locality: Sumatra (west), Sungei Kumbang, Korintji, 4500 ft.

#### Xanthorhoe (Xanthorhoe) gigantis

Prout, 1939

Xanthorhoe (Xanthorhoe) gigantis Prout 1939

##### Materials

- **Type status:** Holotype. **Occurrence:** sex: m; **Record Level:** ownerInstitutionCode: NHM

##### Distribution

Type locality: [West Papua], Mt Goliath

#### Xanthorhoe (Xanthorhoe) hedyphaes

Prout, 1922

Xanthorhoe (Xanthorhoe) hedyphaes Prout 1922b

##### Materials

- **Type status:** Syntype. **Occurrence:** sex: m; **Record Level:** ownerInstitutionCode: NHM

##### Distribution

Type locality: [Moluccas], Ceram [Seram] (central), Manusela, 6000 ft.

#### Xanthorhoe (Xanthorhoe) hyphagna

Prout, 1923

Xanthorhoe (Xanthorhoe) hyphagna Prout 1923

##### Materials

- **Type status:** Holotype. **Occurrence:** sex: m; **Record Level:** ownerInstitutionCode: NHM

##### Distribution

Type locality: Java (west), Badong

#### Xanthorhoe (Xanthorhoe) ludifica

Warren, 1898

Xanthorhoe (Xanthorhoe) ludifica Warren 1898

##### Materials

- **Type status:** Syntype. **Occurrence:** sex: 5m, 3f; **Record Level:** ownerInstitutionCode: NHM

##### Distribution

Type locality: Java, Mt Arjuno

#### Xanthorhoe (Xanthorhoe) nubilosa nubilosa

Warren, 1898

Xanthorhoe (Xanthorhoe) nubilosa nubilosa Warren 1898

##### Materials

- **Type status:** Syntype. **Occurrence:** sex: 1m, 1f; **Record Level:** ownerInstitutionCode: NHM

##### Distribution

Type locality: Java, Mt Arjuno

##### Notes

The species is originally described as subspecies (ab.) of *X. ludifica* Warren (1898)

#### Xanthorhoe (Xanthorhoe) nubilosa klossi

Prout, 1939

Xanthorhoe (Xanthorhoe) nubilosa klossi Prout 1939

##### Materials

- **Type status:** Syntype. **Occurrence:** sex: 2m, 3f; **Record Level:** ownerInstitutionCode: NHM

##### Distribution

Type locality: Sumatra (west), Korintji, 7300 ft., Sungei Kumbang, 10,000 ft.

#### Xanthorhoe (Xanthorhoe) pallida

Rothschild, 1916

Xanthorhoe (Xanthorhoe) pallida Rothschild 1916

##### Materials

- **Type status:** Holotype. **Occurrence:** sex: m; **Record Level:** ownerInstitutionCode: NHM

##### Distribution

Type locality: [West Papua], Snow Mountains, Carstensz Peak, 5000-10,000 ft.

#### Xanthorhoe (Xanthorhoe) pratti

Prout, 1922

Xanthorhoe (Xanthorhoe) pratti Prout 1922b

##### Materials

- **Type status:** Syntype. **Occurrence:** sex: 2m, 2f; **Record Level:** ownerInstitutionCode: NHM

##### Distribution

Type locality: [Moluccas], Ceram [Seram] (central), Manusela, 6000 ft.

#### Xanthorhoe (Xanthorhoe) simplicata

Prout, 1933

Xanthorhoe (Xanthorhoe) simplicata Prout 1933

##### Materials

- **Type status:** Holotype. **Occurrence:** sex: m; **Record Level:** ownerInstitutionCode: NHM

##### Distribution

Type locality: [Moluccas], Buru, Gamoe Mrapat, 5000 ft.

#### Xanthorhoe (Xanthorhoe) succerasina

Prout, 1916

Xanthorhoe (Xanthorhoe) succerasina Prout 1916

##### Materials

- **Type status:** Holotype. **Occurrence:** sex: f; **Record Level:** ownerInstitutionCode: NHM

##### Distribution

Type locality: [West Papua], Mt Goliath, 5000-7000 ft.

#### Xanthorhoe (Xanthorhoe) vulgaris

Rothschild, 1916

Xanthorhoe (Xanthorhoe) vulgaris Rothschild 1916

##### Materials

- **Type status:** Syntype. **Occurrence:** sex: 11m, 19f; **Record Level:** ownerInstitutionCode: NHM

##### Distribution

Type locality: [West Papua], Snow Mountains, 4000-6000 ft.

#### unplaced

#### Acolutha (Emmelesia) pictaria

(Moore 1888)

Acolutha (Emmelesia) pictaria Moore 1888
Acolutha (Emmelesia) pictaria Synonym: *A. pictaria flavifascia*

##### Materials

- **Type status:** Syntype. **Occurrence:** sex: unknown; **Record Level:** ownerInstitutionCode: NHM

##### Distribution

Type locality: India, Darjeeling. Type locality of synonym: [Java (east)], Nongkodjadjar

##### Notes

The species *A. pictaria* is described from India, Darjeeling. The synonym *A. pictaria flavifascia* Prout (1935) is described from [Java (east)], Nongkodjadjar and Singolangoe. The species *A. pictaria* is illustrated in Holloway (1997)

#### Acolutha (Hyria) pulchella pulchella

(Hampson 1891)

Acolutha (Hyria) pulchella pulchella Hampson 1891
Acolutha (Hyria) pulchella pulchella Synonym: *A. pulchella interposita*

##### Materials

- **Type status:** Syntype. **Occurrence:** sex: m; **Record Level:** ownerInstitutionCode: NHM

##### Distribution

Type locality: India, Nilgiri district, S slopes, 3000 ft. Type locality of synonym: Java (east), Nongkodjadjar

##### Notes

The species is described from India, Nilgiri district, S slopes, 3000 ft. The synonym *A. pulchella interposita* Prout (1935) is described from Java (east), Nongkodjadjar

#### Acolutha (Acolutha) subrotunda

Prout, 1922

Acolutha (Acolutha) subrotunda Prout 1922a

##### Materials

- **Type status:** Holotype. **Occurrence:** sex: m; **Record Level:** ownerInstitutionCode: NHM

##### Distribution

Type locality: Lesser Sunda Islands, Sambawa [Sumbawa]

#### Acolutha (Acolutha) talis

Prout, 1928

Acolutha (Acolutha) talis Prout 1928

##### Materials

- **Type status:** Syntype. **Occurrence:** sex: 3f; **Record Level:** ownerInstitutionCode: NHM

##### Distribution

Type locality: Sumatra, Slopes of Mt Korintji, 7500 ft.

#### ‘Asthena’ (Asthena) argyrorrhytes

Prout, 1916

‘Asthena’ (Asthena) argyrorrhytes Prout 1916

##### Materials

- **Type status:** Holotype. **Occurrence:** sex: m; **Record Level:** ownerInstitutionCode: NHM

##### Distribution

Type locality: [West Papua], Mt Goliath, about 139° E, 5000-7000 ft.

##### Notes

The species belong neither to that genus, nor to the tribe Asthenini (Xue and Scoble 2002)

#### ‘Bihastina’ (Asthena) aurantiaca

(Prout 1926)

‘Bihastina’ (Asthena) aurantiaca Prout 1926

##### Materials

- **Type status:** Holotype. **Occurrence:** sex: m; **Record Level:** ownerInstitutionCode: NHM

##### Distribution

Type locality: [West Papua], Mt Goliath, about 139° E, 5000-7000 ft.

##### Notes

The species belong neither to that genus, nor to the tribe Asthenini (Xue and Scoble 2002)

#### Crasilogia (Crasilogia) fulvitincta

Joicey & Talbot, 1917

Crasilogia (Crasilogia) fulvitincta Joicey and Talbot 1917

##### Materials

- **Type status:** Holotype. **Occurrence:** sex: f; **Record Level:** ownerInstitutionCode: NHM

##### Distribution

Type locality: [West Papua], Arfak Mts, Angi Lakes, 6000 ft.

#### Dasimatia (Dasimatia) subusta

Warren, 1898

Dasimatia (Dasimatia) subusta Warren 1898

##### Materials

- **Type status:** Holotype. **Occurrence:** sex: m; **Record Level:** ownerInstitutionCode: NHM

##### Distribution

Type locality: Celebes [Sulawesi], Tawaya, N of Palos Bay

#### Desmoclystia (Desmoclystia) abata

Prout, 1941

Desmoclystia (Desmoclystia) abata Prout 1941

##### Materials

- **Type status:** Syntype. **Occurrence:** sex: 2f; **Record Level:** ownerInstitutionCode: NHM

##### Distribution

Type locality: [West Papua], Mt Goliath, 5000-7000 ft.

#### Desmoclystia (Desmoclystia) abbreviata

Prout, 1941

Desmoclystia (Desmoclystia) abbreviata Prout 1941

##### Materials

- **Type status:** Holotype. **Occurrence:** sex: m; **Record Level:** ownerInstitutionCode: NHM

##### Distribution

Type locality: [West Papua], Mt Goliath, 5000-7000 ft.

#### Desmoclystia (Desmoclystia) aypna

Prout, 1941

Desmoclystia (Desmoclystia) aypna Prout 1941

##### Materials

- **Type status:** Syntype. **Occurrence:** sex: 1m, 3f; **Record Level:** ownerInstitutionCode: NHM

##### Distribution

Type locality: [West Papua], Mt Goliath, 5000-7000 ft.

#### Desmoclystia (Desmoclystia) cnecoplaca

Prout, 1929

Desmoclystia (Desmoclystia) cnecoplaca Prout 1929d

##### Materials

- **Type status:** Holotype. **Occurrence:** sex: m; **Record Level:** ownerInstitutionCode: NHM

##### Distribution

Type locality: [West Papua], Weyland Mountains, Mt Kunupi, 6000 ft.

#### Desmoclystia (Desmoclystia) oniria

Prout, 1941

Desmoclystia (Desmoclystia) oniria Prout 1941

##### Materials

- **Type status:** Syntype. **Occurrence:** sex: 5m, 1f; **Record Level:** ownerInstitutionCode: NHM

##### Distribution

Type locality: [West Papua], Mt Goliath, 5000-7000 ft.

#### Eois (Bardanes) flavata

(Warren 1896)

Eois (Bardanes) flavata Warren 1896

##### Materials

- **Type status:** Syntype. **Occurrence:** sex: 2m, 4f; **Record Level:** ownerInstitutionCode: NHM

##### Distribution

Type locality: Java (west)

#### Eois (Acidalia) impletaria

(Walker 1866)

Eois (Acidalia) impletaria Walker 1866b

##### Materials

- **Type status:** Syntype. **Occurrence:** sex: f; **Record Level:** ownerInstitutionCode: OUM

##### Distribution

Type locality: [West Papua], Misoöl [Misool]

##### Notes

The synonym *E. subrosea* (Warren, 1897) is described from Bali

#### Eois (Eois) ingrataria tambora

Prout, 1923

Eois (Eois) ingrataria tambora Prout 1923

##### Materials

- **Type status:** Syntype. **Occurrence:** sex: 13m; **Record Level:** ownerInstitutionCode: NHM

##### Distribution

Type locality: Lesser Sunda Islands, Sambawa [Sumbawa], Tambora

#### Eois (Hydrelia) pallidula

(Warren 1896)

Eois (Hydrelia) pallidula Warren 1896

##### Materials

- **Type status:** Holotype. **Occurrence:** sex: f; **Record Level:** ownerInstitutionCode: NHM

##### Distribution

Type locality: Java (south), 1500 ft.

##### Notes

The species is illustrated in Holloway (1997)

#### Eois (Pseudoasthena?) plumbacea

(Warren 1894)

Eois (Pseudoasthena?) plumbacea Warren 1894
Eois (Pseudoasthena?) plumbacea Synonym: *E. metriopis*

##### Materials

- **Type status:** Holotype. **Occurrence:** sex: m; **Record Level:** ownerInstitutionCode: NHM

##### Distribution

Type locality: Malaysia, Borneo. Type localities of synonym: Borneo, nr Kalimantan, Pulo Laut

##### Notes

The species is described from Malaysia, Borneo. The synonym *E. metriopis* (Meyrick, 1897) is described from Borneo and nr Kalimantan, Pulo Laut. The species *E. plumbacea* is illustrated in Holloway (1997)

#### Eois (Cretheis) sanguilineata

(Warren 1901)

Eois (Cretheis) sanguilineata Warren 1901b

##### Materials

- **Type status:** Syntype. **Occurrence:** sex: 1m, 1f; **Record Level:** ownerInstitutionCode: NHM

##### Distribution

Type locality: [West Papua], Misoöl [Misool]

#### Eois (Psilocambogia) semirubra

(Warren 1896)

Eois (Psilocambogia) semirubra Warren 1896

##### Materials

- **Type status:** Holotype. **Occurrence:** sex: m; **Record Level:** ownerInstitutionCode: NHM

##### Distribution

Type locality: [West Papua], Humboldt Bay [Yos Sudarso Bay]

#### Eois (Psilocambogia) undulosaria

(Warren 1897)

Eois (Psilocambogia) undulosaria Warren 1897b

##### Materials

- **Type status:** Holotype. **Occurrence:** sex: m; **Record Level:** ownerInstitutionCode: NHM

##### Distribution

Type locality: [Moluccas], Amboina [nr Seram, Ambon]

#### Eois (Eois) verisimilis

Prout, 1922

Eois (Eois) verisimilis Prout 1922a

##### Materials

- **Type status:** Holotype. **Occurrence:** sex: m; **Record Level:** ownerInstitutionCode: NHM

##### Distribution

Type locality: Lesser Sunda Islands, Sambawa [Sumbawa], Tambora, 2500-4000 ft.

#### Lasioedma (Lasioedma) purpureorufa

Rothschild, 1916

Lasioedma (Lasioedma) purpureorufa Rothschild 1916

##### Materials

- **Type status:** Holotype. **Occurrence:** sex: m; **Record Level:** ownerInstitutionCode: NHM

##### Distribution

Type locality: [West Papua], Snow Mountains, Utakwa [Oetakwa] River, 3000 ft.

#### Papuanticlea (Papuanticlea) onaea

Prout, 1939

Papuanticlea (Papuanticlea) onaea Prout 1939

##### Materials

- **Type status:** Syntype. **Occurrence:** sex: 1m, 1f; **Record Level:** ownerInstitutionCode: NHM

##### Distribution

Type locality: [West Papua], Mt Goliath

#### Papuanticlea (Anticlea) subcaesia

(Warren 1903)

Papuanticlea (Anticlea) subcaesia Warren 1903
Papuanticlea (Anticlea) subcaesia Synonym: *P. subcaesia neutralis*

##### Materials

- **Type status:** Syntype. **Occurrence:** sex: 4f; **Record Level:** ownerInstitutionCode: NHM

##### Distribution

Type locality: [Papua New Guinea], Upper Aroa River. Type locality of synonym: [Moluccas], Ceram [Seram], Manusela, 6000 ft.

##### Notes

The species is described from [Papua New Guinea], Upper Aroa River. The synonym *P. subcaesia neutralis* (Prout, 1922) is described from [Moluccas], Ceram [Seram], Manusela, 6000 ft.

#### Papuarisme (Horisme) aeolotis

(Prout 1916)

Papuarisme (Horisme) aeolotis Prout 1916

##### Materials

- **Type status:** Holotype. **Occurrence:** sex: m; **Record Level:** ownerInstitutionCode: NHM

##### Distribution

Type locality: [West Papua], Mt Goliath, 5000-7000 ft.

#### Papuarisme (Horisme) genuflexa

(Prout 1923)

Papuarisme (Horisme) genuflexa Prout 1923

##### Materials

- **Type status:** Syntype. **Occurrence:** sex: 1m, 1f; **Record Level:** ownerInstitutionCode: NHM

##### Distribution

Type locality: [West Papua], Mt Goliath, 5000-7000 ft.

#### Papuarisme (Horisme) illustris

(Prout 1916)

Papuarisme (Horisme) illustris Prout 1916

##### Materials

- **Type status:** Holotype. **Occurrence:** sex: m; **Record Level:** ownerInstitutionCode: NHM

##### Distribution

Type locality: [West Papua], Mt Goliath, 5000-7000 ft.

#### Papuarisme (Horisme) leucotmeta

(Prout 1923)

Papuarisme (Horisme) leucotmeta Prout 1923

##### Materials

- **Type status:** Syntype. **Occurrence:** sex: 3m, 2f; **Record Level:** ownerInstitutionCode: NHM

##### Distribution

Type locality: [West Papua], Mt Goliath, 5000-7000 ft.

#### Diactinia (Euphyia) notata

(Rothschild 1916)

Diactinia (Euphyia) notata Rothschild 1916

##### Materials

- **Type status:** Holotype. **Occurrence:** sex: m; **Record Level:** ownerInstitutionCode: NHM

##### Distribution

Type locality: [West Papua], Snow Mountains, Carstensz Peak, 5000-10,000 ft.

#### Papuarisme (Horisme) symmetrozona

(Prout 1923)

Papuarisme (Horisme) symmetrozona Prout 1923

##### Materials

- **Type status:** Syntype. **Occurrence:** sex: 3m; **Record Level:** ownerInstitutionCode: NHM

##### Distribution

Type locality: [West Papua], Mt Goliath, 5000-7000 ft.

#### Parachaetolopha (Chaetolopha) anomala

(Prout 1941)

Parachaetolopha (Chaetolopha) anomala Prout 1941

##### Materials

- **Type status:** Holotype. **Occurrence:** sex: f; **Record Level:** ownerInstitutionCode: NHM

##### Distribution

Type locality: [West Papua], Mt Goliath, 5000-7000 ft.

##### Notes

The species is described as subspecies of *P. ornatipennis* (Prout, 1941) and illustrated in Schmidt (2002)

#### Parachaetolopha (Parachaetolopha) ferruginoapex

Schmidt, 2002

Parachaetolopha (Parachaetolopha) ferruginoapex Schmidt 2002

##### Materials

- **Type status:** Holotype. **Occurrence:** sex: m; **Record Level:** ownerInstitutionCode: NHM

##### Distribution

Type locality: [West Papua], Mt Goliath, about 139° long., 5000-7000 ft.

#### Parachaetolopha (Chaetolopha) peregrina

(Prout 1929)

Parachaetolopha (Chaetolopha) peregrina Prout 1929b

##### Materials

- **Type status:** Holotype. **Occurrence:** sex: m; **Record Level:** ownerInstitutionCode: NHM

##### Distribution

Type locality: [Moluccas], Ceram [Seram], Manusela, 6000 ft.

##### Notes

The species is described as subspecies of *P. ornatipennis* (Prout, 1941) and illustrated in Schmidt (2002)

#### Parachaetolopha (Parachaetolopha) petasitruncula

Schmidt, 2002

Parachaetolopha (Parachaetolopha) petasitruncula Schmidt 2002

##### Materials

- **Type status:** Holotype. **Occurrence:** sex: m; **Record Level:** ownerInstitutionCode: NHM

##### Distribution

Type locality: [West Papua], Mt Goliath, about 139° long., 5000-7000 ft.

#### Parachaetolopha (Chaetolopha) turbinata

(Prout 1941)

Parachaetolopha (Chaetolopha) turbinata Prout 1941

##### Materials

- **Type status:** Lectotype. **Occurrence:** sex: m; **Record Level:** ownerInstitutionCode: NHM

##### Distribution

Type locality: [West Papua], Mt Goliath, 5000-7000 ft.

##### Notes

Lectotype has been designated (Schmidt 2002)

#### Parapalta (Anapalta) aurifera circumfumata

(Prout 1916)

Parapalta (Anapalta) aurifera circumfumata Prout 1916

##### Materials

- **Type status:** Syntype. **Occurrence:** sex: f; **Record Level:** ownerInstitutionCode: NHM

##### Distribution

Type locality: [West Papua], Mt Goliath, 5000-7000 ft.

#### Parapalta (Anapalta) semiviridis

(Joicey & Talbot 1917)

Parapalta (Anapalta) semiviridis Joicey and Talbot 1917

##### Materials

- **Type status:** Syntype. **Occurrence:** sex: 1m, 1f; **Record Level:** ownerInstitutionCode: NHM

##### Distribution

Type locality: West Irian [West Papua], Wandammen Mts, 3000-4000 ft.

#### Physetobasis (Physetobasis) heliocoma

Meyrick, 1897

Physetobasis (Physetobasis) heliocoma Meyrick 1897

##### Materials

- **Type status:** Holotype. **Occurrence:** sex: f; **Record Level:** ownerInstitutionCode: NHM

##### Distribution

Type locality: Lesser Sunda Islands, Sambawa [Sumbawa]

#### Propithex (Propithex) alternata

Warren, 1899

Propithex (Propithex) alternata Warren 1899a

##### Materials

- **Type status:** Holotype. **Occurrence:** sex: m; **Record Level:** ownerInstitutionCode: NHM

##### Distribution

Type locality: [West Papua], Ron Island

#### Pseudosauris (Syzyx) postfulvata

(Prout 1916)

Pseudosauris (Syzyx) postfulvata Prout 1916

##### Materials

- **Type status:** Holotype. **Occurrence:** sex: m; **Record Level:** ownerInstitutionCode: NHM

##### Distribution

Type locality: [West Papua], Mt Goliath, 5000-7000 ft.

#### Spectrobasis (Spectrobasis) conferens

Prout, 1940

Spectrobasis (Spectrobasis) conferens Prout 1940

##### Materials

- **Type status:** Syntype. **Occurrence:** sex: 2m; **Record Level:** ownerInstitutionCode: NHM

##### Distribution

Type locality: [West Papua], Mt Goliath

#### Spectrobasis (Spectrobasis) impectinata

Prout, 1916

Spectrobasis (Spectrobasis) impectinata Prout 1916

##### Materials

- **Type status:** Holotype. **Occurrence:** sex: unknown; **Record Level:** ownerInstitutionCode: NHM

##### Distribution

Type locality: [West Papua], Mt Goliath, 5000-7000 ft.

#### Sterrhochaeta (Chaetolopha?) antennata

(Warren 1906)

Sterrhochaeta (Chaetolopha?) antennata Warren 1906
Sterrhochaeta (Chaetolopha?) antennata Synonym: *S. viriditincta*

##### Materials

- **Type status:** Holotype. **Occurrence:** sex: m; **Record Level:** ownerInstitutionCode: NHM

##### Distribution

Type locality: [Papua New Guinea], Angabunga River. Type locality of synonym: [West Papua], Snow Mountains, Utakwa [Oetakwa] River, 2500-3000 ft.

##### Notes

The species is described from [Papua New Guinea], Angabunga River. The synonym *S. viriditincta* (Rothschild, 1916) is described from [West Papua], Snow Mountains, Utakwa [Oetakwa] River, 2500-3000 ft.

#### Sterrhochaeta (Sterrhochaeta) aphanisis

Prout, 1941

Sterrhochaeta (Sterrhochaeta) aphanisis Prout 1941

##### Materials

- **Type status:** Holotype. **Occurrence:** sex: f; **Record Level:** ownerInstitutionCode: NHM

##### Distribution

Type locality: [West Papua], Mt Goliath, 5000-7000 ft.

#### Sterrhochaeta (Sterrhochaeta) argyrastrape

Prout, 1916

Sterrhochaeta (Sterrhochaeta) argyrastrape Prout 1916

##### Materials

- **Type status:** Holotype. **Occurrence:** sex: f; **Record Level:** ownerInstitutionCode: NHM

##### Distribution

Type locality: [West Papua], Mt Goliath, 5000-7000 ft.

#### Sterrhochaeta (Sterrhochaeta) biflexa

Prout, 1941

Sterrhochaeta (Sterrhochaeta) biflexa Prout 1941

##### Materials

- **Type status:** Holotype. **Occurrence:** sex: m; **Record Level:** ownerInstitutionCode: NHM

##### Distribution

Type locality: [West Papua], Mt Goliath, 5000-7000 ft.

#### Sterrhochaeta (Sterrhochaeta) lamia

Prout, 1941

Sterrhochaeta (Sterrhochaeta) lamia Prout 1941

##### Materials

- **Type status:** Syntype. **Occurrence:** sex: 1m, 2f; **Record Level:** ownerInstitutionCode: NHM

##### Distribution

Type locality: [West Papua], Mt Goliath, 5000-7000 ft.

#### Sterrhochaeta (Psaliodes?) olivacea

(Rothschild 1916)

Sterrhochaeta (Psaliodes?) olivacea Rothschild 1916

##### Materials

- **Type status:** Holotype. **Occurrence:** sex: m; **Record Level:** ownerInstitutionCode: NHM

##### Distribution

Type locality: [West Papua], Snow Mountains, Utakwa [Oetakwa] River, 3000 ft.

#### Sterrhochaeta (Sterrhochaeta) rectilineata diffidens

Prout, 1941

Sterrhochaeta (Sterrhochaeta) rectilineata diffidens Prout 1941

##### Materials

- **Type status:** Syntype. **Occurrence:** sex: 3f; **Record Level:** ownerInstitutionCode: NHM

##### Distribution

Type locality: [West Papua], Mt Goliath, 5000-7000 ft.

#### Sterrhochaeta (Sterrhochaeta) rectilineata indirecta

Prout, 1958

Sterrhochaeta (Sterrhochaeta) rectilineata indirecta Prout 1958

##### Materials

- **Type status:** Holotype. **Occurrence:** sex: f; **Record Level:** ownerInstitutionCode: NHM

##### Distribution

Type locality: Celebes (west) [Sulawesi], Paloe, Sidaonta, 4500 ft.

#### Sterrhochaeta (Horisme) subtilis

(Prout 1916)

Sterrhochaeta (Horisme) subtilis Prout 1916

##### Materials

- **Type status:** Holotype. **Occurrence:** sex: f; **Record Level:** ownerInstitutionCode: NHM

##### Distribution

Type locality: [West Papua], Mt Goliath, 5000-7000 ft.

#### Visiana (Xanthorhoe) inimica

(Prout 1937)

Visiana (Xanthorhoe) inimica Prout 1937

##### Materials

- **Type status:** Holotype. **Occurrence:** sex: m; **Record Level:** ownerInstitutionCode: NHM

##### Distribution

Type locality: Bali (west), Mondoktoempang, 2500 ft.

##### Notes

The species is described as subspecies of *V. sordidata* (Moore, 1888) and illustrated in Schmidt (2006a), Schmidt (2009)

#### Visiana (Xanthorhoe) robinsoni

(Prout 1939)

Visiana (Xanthorhoe) robinsoni Prout 1939

##### Materials

- **Type status:** Lectotype. **Occurrence:** sex: m; **Record Level:** ownerInstitutionCode: NHM

##### Distribution

Type locality: Sumatra (west), Sungei Kumbang, Korintji, 4500 ft.

##### Notes

The species is described as subspecies of *V. sordidata* (Moore, 1888) and illustrated in Schmidt (2006a). Lectotype has been designated (Schmidt 2006a)

#### Visiana (Xanthorhoe) tamborica

(Prout 1939)

Visiana (Xanthorhoe) tamborica Prout 1939

##### Materials

- **Type status:** Lectotype. **Occurrence:** sex: m; **Record Level:** ownerInstitutionCode: NHM

##### Distribution

Type locality: Lesser Sunda Islands, Tambora, Sambawa [Sumbawa], 2500-4000 ft.

##### Notes

The species is described as subspecies of *V. sordidata* (Moore, 1888) and illustrated in Schmidt (2006a), Schmidt (2009). Lectotype has been designated (Schmidt 2006a)

#### Visiana (Xanthorhoe) ranensis

(Prout 1939)

Visiana (Xanthorhoe) ranensis Prout 1939

##### Materials

- **Type status:** Holotype. **Occurrence:** sex: m; **Record Level:** ownerInstitutionCode: NHM

##### Distribution

Type locality: [Moluccas], Buru, Rana

##### Notes

The species is described as subspecies of *V. vinosa* (Warren, 1907) and illustrated in Schmidt (2013)

## Discussion

The current list presents data on 210 species and 41 subspecies of larentiine moths described from Indonesia so far, of which 33 species occur on Borneo (Malaysia) and were in detail illustrated by Holloway (1997). The Indonesian type specimens are deposited in NHM (239 specimens), OUM (six specimens), NBC (two specimens), and in NHRS, RBINS, ZMMU and ZSM (one specimen each). The majority of species (66%) were described by L.B. Prout, followed by W. Warren (17%). The species and subspecies described from Indonesia belong to seven tribes, namely Asthenini (1.2%), Cidariini (4%), Eupitheciini (38.2%), Larentiini (0.4%), Melanthiini (4%), Trichopterygini (19.1%), Xanthorhoini (10.8%) or have uncertain tribal placement (22.3%).

The tribal placement of many species needs to be evaluated. However, according to preliminary data involving the study of the species described from other regions and apparently occurring in Indonesia (Schmidt, unpubl. data), the tribe Eupitheciini and its close allies seem to be dominant in Indonesia. Moreover, the high-altitude Indonesian fauna is poorly studied so far. Considering high diversity of eupitheciines in mountainous regions and unresolved taxonomic problems in the group (*e.g.* sibling species), a large proportion of Eupitheciini among the larentiine moths is expected.

Many aberrant specimens were reported from Indonesia. For example, Warren (1898) described a form (ab. *incognita*) of *Xanthorhoe ludifica* and later (Warren 1899a) described a form (ab. *atrifasciata*) of *Photoscotosia multiplicata*. Later Prout (1939), Prout (1940), Prout (1941) named forms of *Dysstroma cuneifera* (ab. *integrata*), *D. ceprona* (ab. *russata* and ab. *rufescens*), *Horisme steretica* (ab. *restituta*), *Papuarisme contaminata* (Warren, 1906) (ab. *semipleta*), *Xanthorhoe callisthenes* (ab. *albifusa*) and *X. ludifica* (ab. *incognita*), and described a form of the subspecies *Ardonis filicata mochleutes* (ab. *epacta*) and a form of *Calluga grammophora* (ab. *completa*) (Prout 1958). Although the names of these aberrations are not available nomenclatorially, they may indicate hidden richness of the Indonesian geometrid moth fauna and their existence underpins the necessity of further studies.

The following observations resulted from the study of literature:

- *Ptychotheca pallidivirens* should be transferred to the genus *Bosara* (see Holloway 1997).
- Prout (1941) mentioned “*Desmoclystia prouti* Sick, sp. n.”, without description of the type locality, among other species described from West Papua (Mt Goliath). The type of *D. prouti* without abdomen, as cited in the original description, has never been re-examined.
- Scoble (1999) lists *Eupithecia aspectabilis* Inoue (1996) as described from Indonesia (Maluku [Moluccas]: Aru). However, the type locality of the species is Aru in Pahalgam-Kolohoi in Kashmir, with an altitude of 2800m.
